# Discovering monoacylglycerol lipase inhibitors by a combination of fluorogenic substrate assay and activity-based protein profiling

**DOI:** 10.3389/fphar.2022.941522

**Published:** 2022-08-29

**Authors:** Hui Deng, Qianwen Zhang, Qian Lei, Na Yang, Kai Yang, Jianbing Jiang, Zhiyi Yu

**Affiliations:** ^1^ Department of Respiratory and Critical Care Medicine, West China Hospital, Sichuan University, Chengdu, Sichuan, China; ^2^ Targeted Tracer Research and Development Laboratory, Precision Medicine Key Laboratory of Sichuan Province & Precision Medicine Center, West China Hospital, Sichuan University, Chengdu, Sichuan, China; ^3^ Department of Obstetrics and Gynecology, West China Second University Hospital, Sichuan University, Chengdu, Sichuan, China; ^4^ Department of Medicinal Chemistry, School of Pharmaceutical Sciences, Cheeloo College of Medicine, Shandong University, Jinan, Shandong, China; ^5^ Health Science Center, School of Pharmaceutical Sciences, Shenzhen University, Shenzhen, Guangdong, China

**Keywords:** monoacylglycerol lipase, inhibitor discovery, fluorogenic substrate assay, activity-based protein profiling, anticancer activity

## Abstract

The endocannabinoid 2-arachidonoylglycerol (2-AG) is predominantly metabolized by monoacylglycerol lipase (MAGL) in the brain. Selective inhibitors of MAGL provide valuable insights into the role of 2-AG in a variety of (patho)physiological processes and are potential therapeutics for the treatment of diseases such as neurodegenerative disease and inflammation, pain, as well as cancer. Despite a number of MAGL inhibitors been reported, inhibitors with new chemotypes are still required. Here, we developed a substrate-based fluorescence assay by using a new fluorogenic probe **AA-HNA** and successfully screened a focused library containing 320 natural organic compounds. Furthermore, we applied activity-based protein profiling (ABPP) as an orthogonal method to confirm the inhibitory activity against MAGL in the primary substrate-based screening. Our investigations culminated in the identification of two major compound classes, including quinoid diterpene (**23**, cryptotanshinone) and *β*-carbolines (**82** and **93**, cis- and trans-isomers), with significant potency towards MAGL and good selectivity over other 2-AG hydrolases (ABHD6 and ABHD12). Moreover, these compounds also showed antiproliferative activities against multiple cancer cells, including A431, H1975, B16-F10, OVCAR-3, and A549. Remarkably, **23** achieved complete inhibition towards endogenous MAGL in most cancer cells determined by ABPP. Our results demonstrate the potential utility of the substrate-based fluorescence assay in combination with ABPP for rapidly discovering MAGL inhibitors, as well as providing an effective approach to identify potential targets for compounds with significant biological activities.

## Introduction

The endocannabinoid system (ECS) is a lipid signaling network that regulates a variety of (patho)physiological processes, including anxiety, depression, pain perception, energy balance, appetite control, and inflammation (Pagotto et al., 2006; Di Marzo, 2011; Nomura et al., 2011; Haugh et al., 2016). In general, ECS is constituted of three parts: 1) the cannabinoid receptors (CB1R and CB2R) (Munro et al., 1993), known as G-protein-coupled receptors (GPCR); 2) endocannabinoids (eCBs), agonists of the cannabinoid receptors (Iannotti et al., 2016) [7]; and 3) biosynthetic and metabolic enzymes of the eCBs (Blankman et al., 2007; Powell et al., 2015). Among them, 2-arachidonoylglycerol (2-AG) and anandamide (AEA) are the most abundant endocannabinoids that activate CB1R and CB2R, modulating neurotransmission and immune responses (Devane et al., 1992; Hanus et al., 2001; Murataeva et al., 2014). The degradation of eCBs is attributable to different enzymatic pathways, such as fatty acid amide hydrolase (FAAH)-involved AEA hydrolysis, as well as 2-AG inactivation by monoacylglycerol lipase (MAGL) and two additional serine hydrolases, *α/β* hydrolase domain 6 and 12 (ABHD6 and ABHD12) (Hanus et al., 2001; Blankman et al., 2007; Savinainen, 2012; Cao et al., 2019). As ECS is a promising therapeutic target, early efforts of drug discovery focused on direct pharmacological intervention of ECS by various agonists and antagonists of the cannabinoid receptors, such as the selective CB1R antagonist rimonabant and the agonist tetrahydrocannabinol (THC) (Ortar et al., 2008; Pacher and Kunos, 2013). However, the concomitant psychiatric adverse effects (e.g., depression and suicide) have limited their use as therapeutic agents. To avoid this issue, an indirect approach by targeting the enzymes (e.g., MAGL and FAAH) that regulate eCB levels has emerged as an alternative strategy for drug discovery (Long et al., 2009; Mulvihill and Nomura, 2013; Ignatowska-Jankowska et al., 2014; Deng and Li, 2020a).

Among them, MAGL is a membrane-associated soluble enzyme, which hydrolyses the majority of brain 2-AG (∼85%) into arachidonic acid (AA) and glycerol, while other 2-AG hydrolases such as ABHD6 and ABHD12 contribute to less than 20% of 2-AG hydrolysis (Savinainen, 2012). Studies have shown that inhibition of MAGL activity not only induces the elevation of 2-AG levels but also reduces the amount of AA, which is the crucial precursor of pro-inflammatory eicosanoids, for example, prostaglandins (e.g., PGE2 and PGD2) (Long et al., 2009). Therefore, inactivation of MAGL may have the potential values for therapeutic responses such as attenuating neuroinflammatory (Alhouayek et al., 2013). Moreover, MAGL is found highly expressed in aggressive human cancer cell lines and primary tumors, where it regulates a pro-tumorigenic signaling network of lipids that drives cancer cell migration, invasion, survival, and tumor growth (Nomura et al., 2010). As such, inhibition of MAGL activity may generate a therapeutic effect on cancer diseases (Shah et al., 2021). Overall, MAGL is a very promising therapeutic target, either from the cannabinoid signaling-dependent pathway or from the physiological role of MAGL itself. Based on these, MAGL inhibitors can be developed as possible therapeutic agents in the treatment of various diseases such as neurodegenerative disease, inflammation, pain, as well as cancer (Nomura et al., 2010; Taschler et al., 2011; Chen et al., 2012).

To date, a variety of MAGL inhibitors were developed, such as carbamates, ureas, maleimides, disulfides, and so on (Deng and Li, 2020b). Among them, the covalent, irreversible inhibitors, for example, the piperidine carbamates (e.g., JZL184 and KML29), are the most studied ones by forming a covalent bond with the reactive serine residue in the active site (Long et al., 2009; Ignatowska-Jankowska et al., 2014; Deng and Li, 2020b). However, the prolonged inhibition of MAGL by these irreversible agents induces CB1R desensitization, a loss of cannabinoid-mediated effects, and physical dependency (Schlosburg et al., 2010). Thus, the discovery of MAGL reversible inhibitors becomes a possible trend to avoid the functional antagonism of CB1R. Although a couple of MAGL reversible inhibitors have been reported, most of them are based on similar motifs (e.g., with a piperidine or piperazine ring linked to an amide group). Moreover, most of the present inhibitors are not completely selective against MAGL or/and do not possess pharmacokinetic properties to act as good drug candidates or selective tools to interpret the biological functions of MAGL in (patho)physiological process. Therefore, there is still an unmet need to identify a novel scaffold for developing MAGL inhibitors.

**FIGURE 1:**
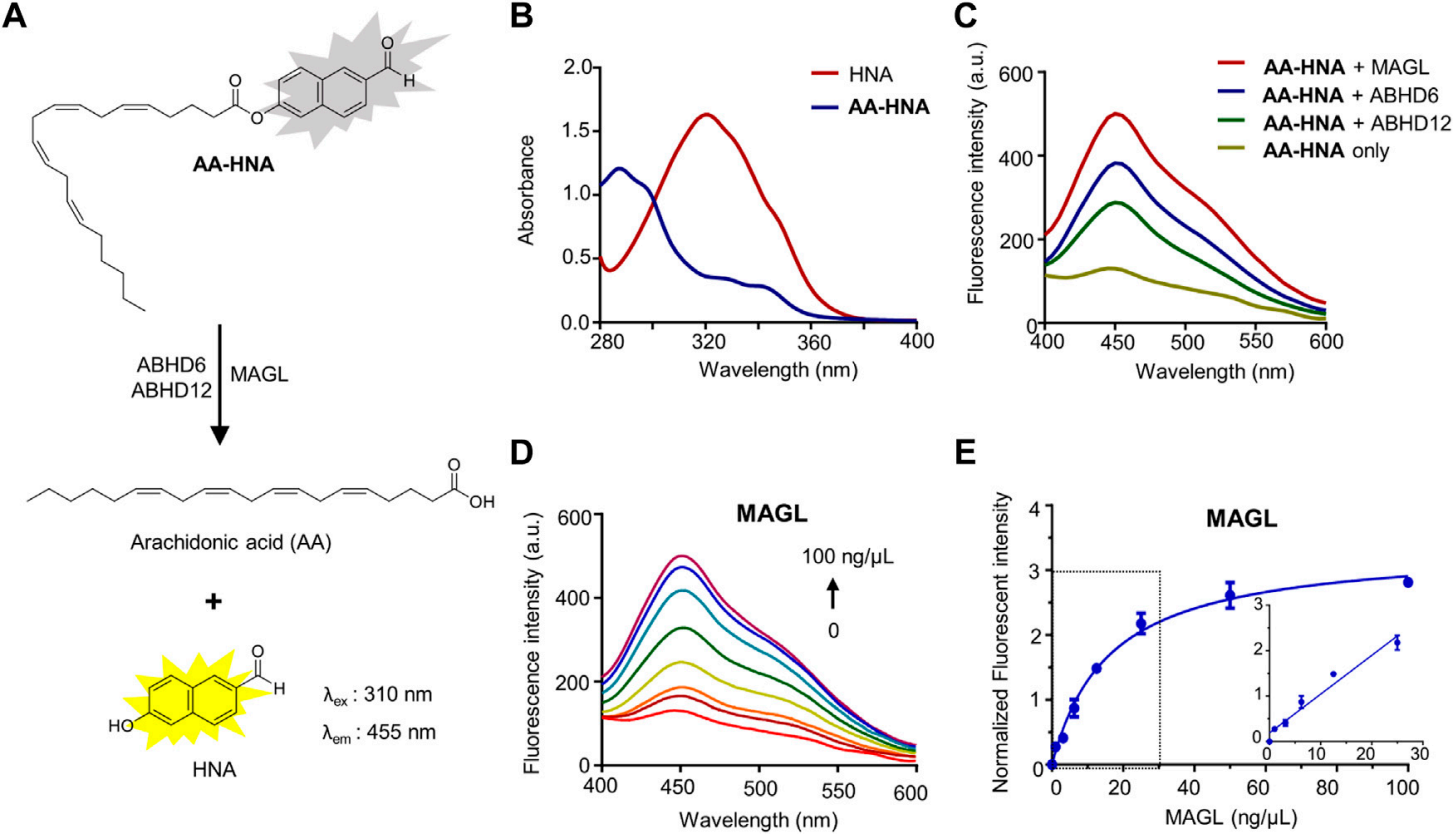
Fluorescence turn-on responses of **AA-HNA** on 2-AG hydrolases (MAGL, ABHD6, and ABHD12). **(A)** Proposed mechanism of **AA-HNA** catalyzed by 2-AG hydrolases (MAGL, ABHD6, and ABHD12). **(B)** Absorbance spectra of **AA-HNA** and HNA. **(C)** Fluorescence spectra response of **AA-HNA** after incubation (30 min) with MAGL, ABHD6, or ABHD12. **(D)** Concentration-dependent fluorescence spectra response at the emission of 455 nm with the increasing of MAGL concentration (0–100 ng/μl) in the presence of **AA-HNA** (10 µM). **(E)** Titration curve of **AA-HNA** (10 µM) with increasing concentration of MAGL (0–100 ng/μl), and the linear relationship was observed in the range of 0–25 ng/μl.

To identify new lead compounds acting as MAGL inhibitors, high-throughput screening is one of the general strategies, and a rapid, effective, as well as the low-cost assay, is crucial for screening a compound library. A common screening method often requires an accurate biochemical readout of enzyme activity and robust assay reproducibility. Therefore, we set out to develop a reliable method to screen and characterize MAGL inhibitors. As fluorescent probes have currently been used for drug candidate screening with a couple of advantages such as easy operation, highly sensitive, and real-time detection (Tian et al., 2020; Dou et al., 2022), we set out to develop a substrate-based fluorescent assay for the evaluation of MAGL activity. In this study, we synthesized a new fluorescent probe (**AA-HNA**) by introducing AA as a specific recognition moiety for MAGL into the scaffold of a fluorescent group 6-hydroxy-2-naphthaldehyde (HNA) (Figure 1A, Supplementary Scheme S1). As **AA-HNA** serves as a surrogate substrate for MAGL, we developed a biochemical assay using **AA-HNA** with recombinant MAGL obtained from HEK293T cells overexpressing MAGL. In the end, we assembled a library of ∼320 natural organic compounds with potential therapeutic properties (e.g., anti-cancer and anti-inflammation) and screened the library with the optimal assay condition to identify MAGL inhibitors with new chemotypes.

During the process of MAGL inhibitor discovery, a comprehensive selectivity profile is necessary, particularly the selectivity over enzymes linked in 2-AG and AEA hydrolysis (e.g., ABHD6, ABHD12, and FAAH). In addition, as MAGL belongs to serine hydrolases, a superfamily with more than 200 enzymes that use serine as the common active site, a family-wide selectivity profile of MAGL inhibitors over other serine hydrolases is important. Recently, activity-based protein profiling (ABPP) is a highly useful chemical biological technique to assess the activity and selectivity of serine hydrolase inhibitors in a complex native system (Cravatt et al., 2008). Yet, to the best of our knowledge, there is a limited description of the combination of ABPP with high-throughput screening assays to evaluate inhibitor activity and selectivity in the earliest stage of lead identification. Here, we combined our substrate-based fluorescent assay and ABPP to identify MAGL inhibitors with new chemotypes from a natural organic compound library.

## Materials and methods

### General remarks

The common reagents were purchased from commercial sources. KML29 was purchased from Selleckchem with ≥98% purity. DO264 and KT182 were from MedChemExpress with ≥98% purity. All buffers, as well as solutions, were prepared using analytical grade reagents or solvents, as well as Millipore water (deionized using a Millio A10 BiocelTM, with a 0.22 µm filter). Buffers are prepared at room temperature and stored at 4°C, unless stated otherwise. The antibodies presented in this study, including ABHD6 (#97573), MAGL (#abs77398), and ABHD12 (#abs180944) were purchased from Cell Signaling Technology and Absin, respectively. Electrophoresis reagents were from Bio-Rad Laboratories. Cell reagents and culture plates were purchased from Celbio and BIOFIL, respectively. All fluorescence analyses were conducted on the BioTek microplate reader and Horiba Jobin Yvon-Edison Fluoromax-4. NMR spectra were recorded on a Bruker AV spectrometer at 400 (^1^H) and 101 (^13^C) MHz using CDCl_3_ as solvent. High‐resolution mass spectra (HRMS) were recorded on a Thermo Scientific LTQ Orbitrap XL. HPLC purification was performed on a preparative LC-MS system with an Agilent 6,110 or 6,120 mass spectrometer detector (Agilent 1,200 series). Graphs and statistics were performed with GraphPad Prism 6, and Bio-Rad Image-lab was used for gel analysis and quantification.

### Synthesis of the probe AA-HNA

The starting material of 6-hydroxy-2-naphthaldehyde (HNA) and arachidonic acid (AA) were commercially available. The fluorogenic substrate AA-HNA was prepared as follows: to the solution of arachidonic acid (335 ml, 1.1 mmol) and DMAP (12.2 mg, 0.1 mmol) in CH_2_Cl_2_ (10 ml) was added HNA (172 mg, 1.0 mmol) in CH_2_Cl_2_, followed by the dropwise addition of dicyclohexylcarbodiimide (228 mg, 1.1 mmol) in 10 ml CH_2_Cl_2_. The reaction mixture was stirred for 12 h at room temperature. When the reaction was completed and the mixture was diluted with water, and then extracted with ethyl acetate. The combined organic layer was washed with water, and brine, dried over MgSO_4_, and concentrated under reduced pressure. The crude product was purified by column chromatography on silica gel to afford AA-HNA (329 mg, 72%). HRMS [ESI^+^] m/z: calculated for C_31_H_38_O_3_ [M + H]^+^ 459.2899, found: 459.3555.^1^H-NMR (400 MHz, CDCl_3_): δ 10.13 (s, 1H), 8.31 (s, 1H), 8.00 (d, *J* = 8.8 Hz, 1H), 7.95 (d, *J* = 9.9 Hz, 1H), 7.87 (d, *J* = 8.5 Hz, 1H), 7.62 (d, *J* = 2.2 Hz, 1H), 7.33 (dd, *J* = 8.9, 2.3 Hz, 1H), 5.51–5.30 (m, 8H), 2.88–2.79 (m, 6H), 2.65 (t, *J* = 7.5 Hz, 2H), 2.25 (dd, *J* = 13.5, 7.1 Hz, 2H), 2.05 (q, *J* = 7.0 Hz, 2H), 1.89 (p, *J* = 7.5 Hz, 2H), 1.39–1.26 (m, 6H), 0.88 (t, *J* = 6.9 Hz, 3H). ^13^C-NMR (101 MHz, CDCl_3_) δ 192.37, 172.32, 151.30, 137.49, 134.54, 134.39, 131.47, 130.95, 130.92, 129.70, 129.19, 129.07, 129.04, 128.76, 128.48, 128.22, 127.92, 123.97, 122.94, 119.32, 34.16, 31.94, 29.75, 27.65, 26.93, 26.10, 26.09, 26.07, 25.11, 23.01, and 14.53.

### Cell culture

Various cancer cell lines, including human colon cancer cell line HT-29, ovarian cell line OVCAR-3, melanoma cell line B16-F10, skin squamous cancer cells A431, cervical cancer cells Hela, lung cancer cell lines A549, H1975, and HCC827, were purchased from American type culture collection (ATCC). A431, H1975, B16-F10, OVCAR-3, HT-29, HCC827, Hela, and A549 cells were cultured in Dulbecco’s modified Eagle medium (DMEM, Gibco) containing 10% fetal bovine serum (FBS, Gibco), penicillin (100 U/ml) and streptomycin (100 μg/ml). All cell lines were maintained in a humidified 37°C incubator with 5% CO_2_. The medium was refreshed every 2–3 days and the cells were passaged at ∼90% confluence by adding the fresh medium, followed by vigorously pipetting the resuspend cells. Of note, the cells were only washed with PBS when a significant number of dead cells were observed.

### Preparation of membrane fractions overexpressing MAGL/ABHD6/ABHD12

HEK293T cells were cultured at 37°C under 5% CO_2_ in a DMEM medium with stable glutamine and phenolred, containing 10% fetal bovine serum (FBS, Gibco), penicillin, and streptomycin. The cells were passaged every 2–3 days by resuspension in the medium at ∼90% confluence. The membranes used in this study were prepared from transiently transfected HEK293T cells. Briefly, 1 day prior to transfection, ∼10^7^ cells were seeded in 15 cm plates, and then the cells were transfected by the addition of a mixture of polyethyleneimine and plasmid DNA (3:1, 60, and 20 µg) in 2 ml of medium (without serum). Of note, the empty pcDNA3.1 vector was used in the Mock-control. After 24 h, the medium was removed and the cells were refreshed with fresh medium. After 72 h, the cells were harvested by suspending in 20 ml of medium, and the supernatant was removed by centrifugation for 10 min at 1,000 rpm. The cell pellet was flash frozen in liquid nitrogen and stored at −80°C until use. Subsequently, the cell pellets were suspended in lysis buffer (30 mM Tirs, pH 7.5, 1 mM MgCl_2_, 25 U/ml Benzonase), which was homogenized by sonication (3 × 10 bursts) and incubated for 30 min on ice. The membrane fractions overexpressing MAGL/ABHD6/ABHD12 were separated by ultracentrifuge (100,000 g, 30 min, 4 C). The protein concentration was determined with a Qubit protein assay (Invitrogen). The protein samples were quickly frozen in liquid nitrogen and stored at −80 C until use.

### MAGL activity assay

The MAGL activity assay is based on the hydrolysis of 2-arachidonoylglycerol-based fluorogenic substrate by MAGL-overexpressing membrane preparations from transiently transfected HEK293T cells. In brief, the reactions were performed in HEPES buffer (40 mM HEPES, pH 7.5, 0.1 mg/ml BSA) in black, flat bottom 96-well plates. The final protein concentration of membrane fractions from overexpressing human MAGL HEK293T cells was 12.5 μg/ml 5 µl of inhibitors were added from×40 concentrated DMSO stocks in the 96-well plate containing 145 µl assay buffer, followed by the addition of 40 µl of the protein (12.5 μg/ml). After the incubation of the inhibitor-protein complex for 30 min, 10 µl of the fluorogenic substrate (AA-HNA, 200 µM) was added and fluorescence was measured in 1 min intervals for 30 min on a plate reader (BioTek). Final assay concentrations: 12.5 μg/ml MAGL, 200 μM AA-HNA, and 5% DMSO in the total volume of 200 µl. All the measurements were performed in N = 2, n = 2 for inhibitors and N = 2, n = 4 for controls with Z′ value ≥0.6. The slope is determined in 5–15 min for all the experiments.

### ABHD6 activity assay

The ABHD6 activity assay is based on the same principle as the MAGL activity assay described earlier, but with human ABHD6 overexpressing membrane preparations at a final protein concentration of 50 μg/ml and 200 µM AA-HNA. Other conditions are the same as those of the MAGL activity assay.

### Determination of ABHD12 activity using activity-based protein profiling

The ABHD12 activity assay is based on competitive activity-based protein profiling (ABPP) with human ABHD12 overexpressing membrane fractions. Briefly, the membrane proteome (1 mg/ml, 20 µl) was incubated at room temperature with 0.5 µl DMSO (vehicle) or inhibitor for 30 min. Subsequently, the samples were treated with the activity-based probe FP-TAMRA (250 nM, final concentration) for 20 min. The reactions were then quenched by 10 µl sample buffer (×4) (final concentrations: 60 mM Tris, pH 6.8, 2% (w/v) SDS, 10% (v/v) glycerol, 5% (v/v) β-mercaptoethanol, 0.01% (v/v) bromophenol blue). The samples were directly loaded and resolved on SDS-PAGE gel (10% acrylamide), which were then scanned using a ChemiDoc MP system (Cy3 settings, BioRad).

### Mouse membrane preparation

Mouse brains were isolated according to guidelines approved by the ethical committee of Western China Hospital, Sichuan University (No. 2021765A). Mouse brains were dounce homogenized lysis buffer (20 mM HEPES, 2 mM DTT, 1 mM MgCl_2_, 25 U/mL benzonase) and incubated on ice for 15 min, followed by low-speed spin (2,500 g, 3 min, 4 C) to remove debris. The supernatant was subjected to ultracentrifugation (100,000 g, 45 min, 4°C) to generate the cytosolic fraction in the supernatant, as well as the membrane fraction as a pellet. After removal of the soluble supernatant, the pellet was resuspended in storage buffer (20 mM HEPES, 2 mM DTT) by pipetting. Total protein concentrations in membrane and soluble fractions were determined using a Qubit protein assay (Invitrogen). The obtained samples were stored at −80°C until further use.

### Competitive ABPP selectivity assay

The competitive ABPP selectivity assay was performed according to the previously reported protocol. Briefly, mouse brain membrane proteome (2 mg/ml, 20 µl) was preincubated for 30 min with 0.5 µl of DMSO (vehicle) or inhibitor (100 μM, finial concentration). Subsequently, the proteome-inhibitor complex was treated with the activity-based probe FP-TAMRA (250 nM, final concentration) for 20 min. The reactions were then quenched by 10 µl sample buffer (×4). The obtained samples were directly loaded and resolved on SDS-PAGE gel (10% acrylamide). The gels were then scanned and analyzed using a ChemiDoc MP system (Cy3 settings, BioRad). All the samples were measured and duplicated.

### Western blot

Western blot experiments were performed to investigate the expression levels of MAGL, ABHD6, and ABHD12 in various cancer cells according to the previously reported protocols. In brief, cancer cells were washed with phosphate-buffered saline (PBS), harvested by using a cell scraper, and collected by centrifugation. Cell pellets were washed with PBS and lysed with RIPA buffer (Thermo Scientific, #89900) on ice for 15 min. After sonication, the protein concentration was determined by Qubit protein assay (Invitrogen) and normalized to 2 mg/ml. Subsequently, 20 µl of protein sample was loaded to each lane in the gel and resolved on an SDS-PAGE gel (10% acrylamide). After that, the protein samples were transferred from gel to a polyvinyl difluoride (PVDF) membrane using a Trans-Blot Turbo (BioRad). The PVDF membranes were then blocked with BSA blocking buffer for 2 h at room temperature, followed by incubation with different primary antibodies (4°C, overnight), including MAGL (#abs77398), ABHD6 (#97573), and ABHD12 (#abs180944). The membranes were washed with TBST buffer 3 times and then incubated with anti-rabbit IgG (HRP-linked antibody, #7074) for 1 h at room temperature. In the end, the blots were washed in PBST and immunoreactive proteins were detected using a luminal solution with ECL enhancer and H_2_O_2_ by ChemiDoc MP (BioRad).

### Cell viability assay

The antiproliferation activity of the compounds against the cancer cell lines was evaluated using Cell Counting Kit-8. Briefly, cells (∼1,000 per well) in 100 µl of culture medium were plated in a 96-well plate for 24 h and 100 µl of medium with different concentrations of the tested compounds was added to each well for 48 h. Then 10 μl of CCK8 solution (#CK04, Dojindo) was added and incubated at 37°C for 2 h. The absorbance of each well was measured with a plate reader (BioTeck) at 450 nm. Cell viability was calculated as follows: cell viability (%) = [(As-Ab)/(Ac-Ab)] × 100. Among them, As represents the absorbance of experimental wells, Ab represents the absorbance of blank wells and Ac represents the absorbance of control wells. The experiments were repeated at least three times and IC_50_ values were determined by plotting a log(inhibitor) v. s normalized response (Variable slope) dose–response curve generated using GraphPad Prism software.

### Screening cancer cell lines with ABPP

To identify the potential off-targets of the compounds with anti-cancer activities, ABPP was applied. Cancer cells were seeded in the dishes and were washed with PBS, scraped, centrifugated (3,000 × g, 3 min) and pellets snap frozen in liquid in nitrogen. For ABPP experiments, the cell pellets were lysed by probe sonication (3 × 10 bursts) in PBS, followed by the determination of protein concentration of the intact cell lysate by Qubit assay. Subsequently, 20 µl of the cell lysate (1 mg/ml) was preincubated with 0.5 µl of DMSO (vehicle) or inhibitor (100 μM, finial concentration) for 30 min, followed by the treatment with FP-TAMRA (500 nM, final concentration) for 20 min. The reactions were then quenched by 10 µl sample buffer (×4). The obtained samples were directly loaded and resolved on SDS-PAGE gel (10% acrylamide). The gel-based ABPP results were then scanned and analyzed using a ChemiDoc MP system (Cy3 settings, BioRad).

### Molecular docking

The X-ray crystal structure of the human MAGL-compound **3i** complex (PDB code: 5ZUN, https://www.rcsb.org/structure/5ZUN) was selected for molecular docking studies. The PDBQT files for the MAGL and compound **23** were prepared using AutodockTools 1.5.6. To prepare the PDBQT files for docking, essential hydrogen atoms and Kollman united atom charges were added using AutoDock Tools (Trott and Olson, 2010). Then, the docking studies were performed using the AutoDock Vina program package (version 1.1.2). A grid box of size 22.7 × 16.4 × 20.8 points with a grid spacing of 0.375 Å was generated using AutoGrid. The grid was centered at x, y, and z coordinates of −13.1, 21.4, and −10.5, respectively, which was derived from the binding of compound **3i** according to its crystal structure. The post docking analysis was conducted using PyMOL software (Schrödinger, L. and DeLano, W, 2020. PyMOL; Available at: http://www.pymol.org/pymol). The 2D ligand-protein interaction diagram was generated using the LigPlot^+^ program (Laskowski and Swindells, 2011).

## Results

### Spectral properties of the substrate-based fluorogenic probe AA-HNA

First, we evaluated the spectral properties of **AA-HNA**, including UV absorption and fluorescence spectra. As shown in Figure 1, **AA-HNA** had minimal absorption at 330 nm, whereas HNA showed a strong absorption within the same wavelength (Figure 1B). As expected, **AA-HNA** exhibited a low fluorescent signal with the excitation of 330 nm, whereas it generated a significant fluorescent signal after the addition of recombinant human MAGL (Figure 1C). The remarkable fluorescence enhancement of **AA-HNA** at the emission of 455 nm in the presence of MAGL indicated the hydrolysis of **AA-HNA** by MAGL. Because ABHD6 and ABHD12 are also capable to catalyze 2-AG hydrolysis, we next examined the sensitivity of **AA-HNA** hydrolysis by human ABHD6 and ABHD12. Not surprisingly, we observed an increase of fluorescent intensity at 455 nm in the presence of ABHD6, as well as ABHD12 (Figure 1C). However, the enhancement window of fluorescent intensity for ABHD6 and ABHD12 was much lower than that of MAGL, particularly for ABHD12 with only ∼2-fold of fluorescent enhancement. Furthermore, we investigated whether the fluorogenic reaction of **AA-HNA** by 2-AG hydrolases was protein concentration dependency. For MAGL, a clear concentration-dependent fluorescence enhancement was observed and the linear range was from 0 to 25 ng/μl (Figures 1D,E). The hydrolysis of **AA-HNA** by ABHD6 was also observed as protein concentration-dependent, while the concentration-dependent hydrolysis of **AA-HNA** by ABHD12 was less significant (Supplementary Figure S1), implicating the low enzymatic activity of ABHD12 towards **AA-HNA**. Moreover, a time-dependent increase of fluorescence intensity in the presence of MAGL and **AA-HNA** was observed with the excitation of 330 nm and emission of 455 nm (Supplementary Figure S2). Similar effects were observed for ABHD6 and ABHD12, but ABHD12 showed lower fluorescence signals when compared with MAGL and ABHD6 (Supplementary Figure S2), further confirming the low enzymatic activity of ABHD12 towards **AA-HNA** hydrolysis.

### Development of screening assays based on the substrate-based fluorogenic probe AA-HNA

**FIGURE 2:**
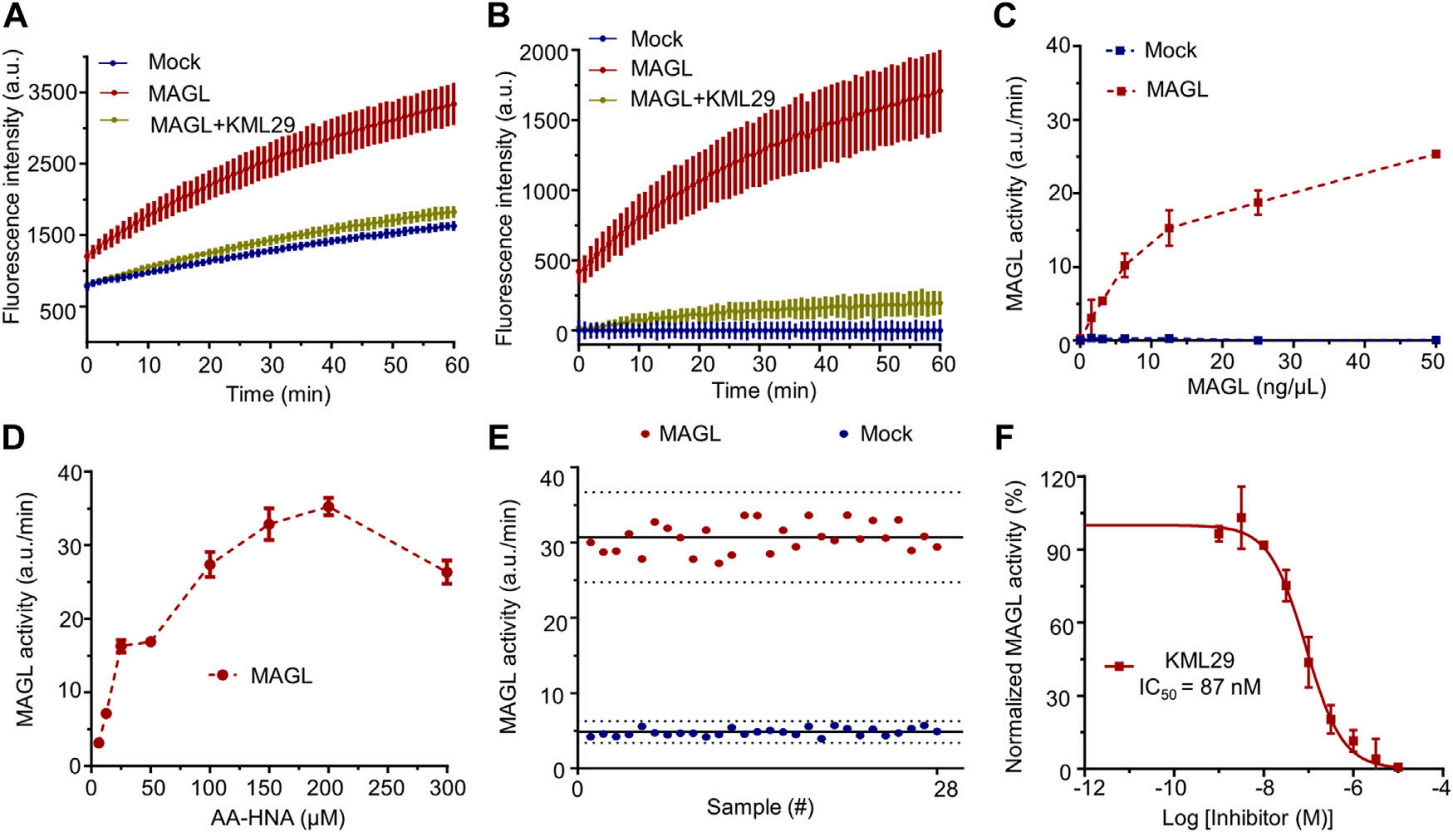
Setup and optimization of **AA-HNA**-based activity assay for MAGL. **(A)** Time course of **AA-HNA** hydrolysis by MAGL (50 ng/μl), resulting in an increase of fluorescence over time. Preincubation with MAGL inhibitor KML29 (1 µM) resulted in the reduction of the fluorescent signal. Membrane proteins from Mock-transfected cells served as a negative control. **(B)** Time course of 2-AG hydrolysis by MAGL, corrected for background fluorescence of the Mock-transfected negative control. **(C)** Optimization of protein concentration for the MAGL activity assay. MAGL activity was monitored in time with various concentrations of MAGL. Notably, MAGL activity was determined from the slope in the linear region (5–15 min) of the specific MAGL activity. **(D)** Optimization of **AA-HNA** concentration for the MAGL activity assay. **(E)** Z′-factor data plot of **AA-HNA**-based MAGL activity assay. The solid lines represent the mean slope of individual data points and dashed lines represent the SD values above and below the mean. The measurement was performed in n = 28. Note that MAGL was used as positive control (mean ± SD = 30.66 ± 1.97) and Mock-membranes were used as negative controls (mean ± SD = 4.80 ± 0.50). Z′-factor and signal to background (S/B) ratio were determined as 0.71 and 6.39, respectively. **(F)** Dose-response curve and IC_50_ determination of MAGL inhibitor KML29. Proteins were incubated with DMSO (vehicle) or various concentrations of the inhibitor for 30 min at room temperature. Data were corrected for background fluorescence observed for Mock-transfected membranes treated with DMSO. The corrected positive control of MAGL activity was normalized as 100%, and the obtained percentage values of MAGL activity were then subjected to a non-linear dose-response analysis with variable slope. The experiments were performed in N = 2, n = 2/4 for controls, with Z′-factor ≥ 0.6. Data represent means ± SEM.

First, the human recombinant MAGL and ABHD6 were prepared from membranes of HEK293T cells overexpressing MAGL and ABHD6 (Supplementary Figure S10), respectively. As shown in Figure 2A, the hydrolysis of **AA-HNA** by membrane fractions of HEK293T cells with human MAGL overexpression was in a time-dependent increase in fluorescent signal. Preincubation of MAGL with KML29 (a known MAGL inhibitor, 1 µM) significantly reduced the fluorescent signal to the background level. In a similar fashion, we also observed the fluorescent signal was time-dependent in the ABHD6 assay (Supplementary Figure S3), and that was blocked by incubation with a selective ABHD6 inhibitor KT185 (10 µM). Of note, a small fluorescence signal window was observed between recombinant ABHD12 and Mock-transfected cells (Supplementary Figure S4), suggesting **AA**-**HNA** is not suitable for developing an activity assay for ABHD12. After correction by background fluorescence from Mock-membranes, a significant signal window between MAGL and Mock-membrane fractions was observed (Figure 2B). A similar result was observed between ABHD6 and Mock after background signal correction (Supplementary Figure S3). To optimize the conditions of the biochemical assay, we started with protein concentration optimization. Of note, the slope in the linear region of corrected fluorescence measurements was applied to determine the enzyme activity. As shown in Figure 2C, the optimal concentration for membrane proteins overexpressing MAGL was determined as 12.5 ng/μL. For ABHD6, the optimal concentration was 50 ng/μl (Supplementary Figure S3). To optimize the concentration of **AA-HNA**, a linear correlation between enzymatic activity (MAGL/ABHD6) and **AA-HNA** concentration was observed up to 200 µM (Figure 2D, Supplementary Figure S3). Subsequently, the assay buffer condition was also investigated and the optimal condition was in a HEPES buffer (40 mM HEPES, pH 7.5, 0.1 mg/ml BSA) (Supplementary Figure S5). Finally, we obtained a Z′-factor data plot to evaluate the **AA-HNA**-based MAGL assay, which resulted in an S/B ratio of 6.39 and Z′-factor of 0.71 (Figure 2E). These results suggest that the **AA-HNA**-based assay is capable to screen inhibitors against MAGL. To validate the accuracy of the assay, we selected KML29 to generate a dose-response curve, which resulted in an IC_50_ value of 87 nM (Figure 2F), while the literature reported IC_50_ value for KML29 is in the range of 2.5–95 nM (Aaltonen et al., 2013; Deng and Li, 2020b).

### Screening a compound library to discover MAGL inhibitors

**FIGURE 3:**
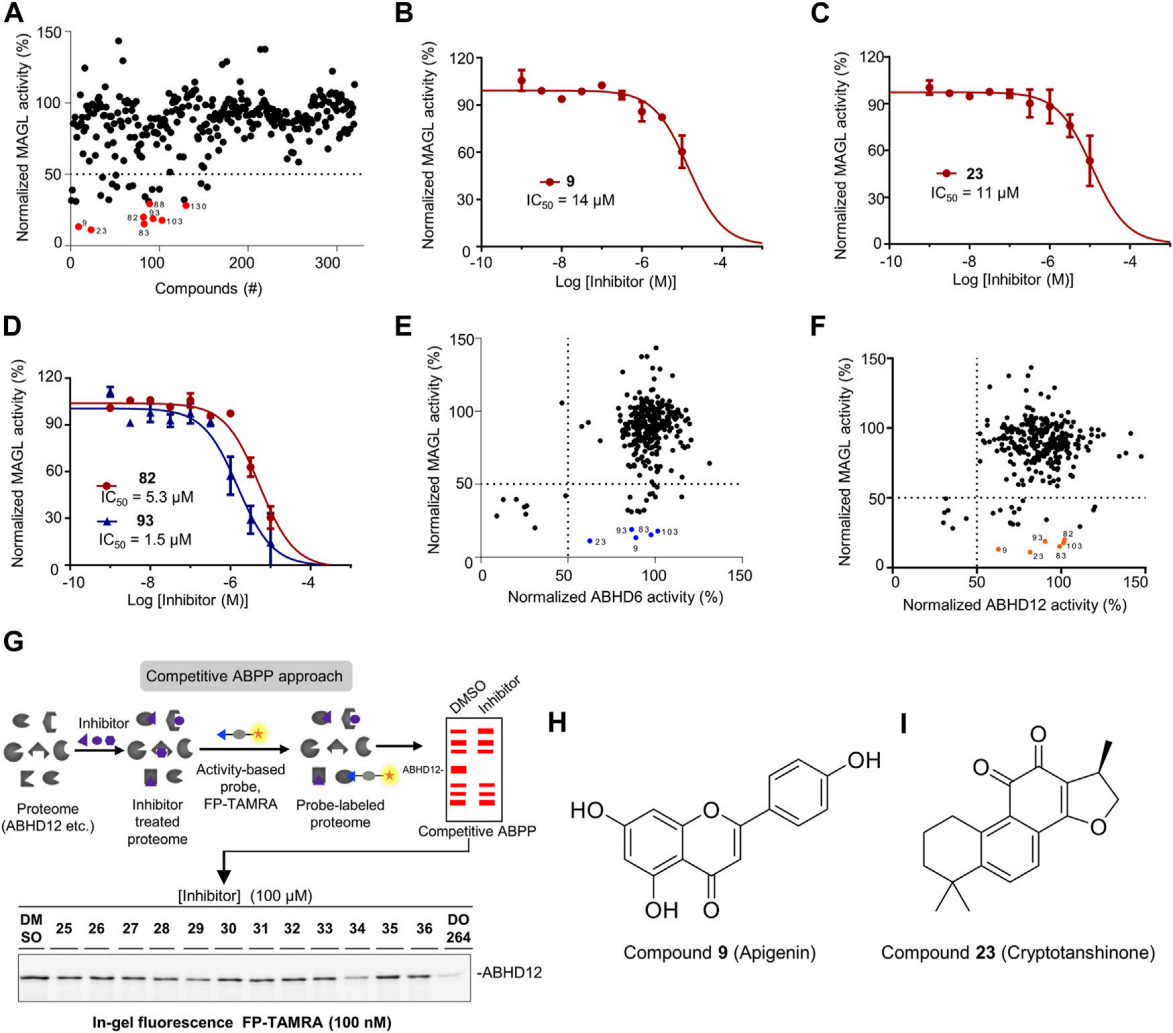
Screening the focused library of natural organic compounds to identify MAGL inhibitors selective over ABHD6 and ABHD12. **(A)** A total of 320 compounds were screened in duplicate (N = 2) at 100 µM using the **AA-HNA**-based assay against MAGL. Among them, 26 compounds showed >50% inhibitory activity and 8 compounds (indicated in red) showed >70% inhibitory activity and were selected for further concentration-response analysis. **(B–D)** Concentration-dependent inhibition of MAGL activity by compounds **9 (B)**, **23 (C)**, **82** and **93 (D)** as measured by the **AA-HNA** assay. Data represent means ± SEM, n = 4 per group. **(E)** Analysis of MAGL inhibition vs. ABHD6 inhibition (100 µM compounds). **(F)** Analysis of MAGL inhibition vs. ABHD12 inhibition (100 µM compounds). **(G)** Representative gels of competitive activity-based protein profiling (ABPP) show the evaluation of ABHD12 activity using activity-based probe FP-TAMRA (250 nM). **(H,I)** Chemical structures of compounds **9** and **23**.

Having optimized the assay conditions for MAGL, we set out to screen a compound library containing 320 natural organic compounds with potential therapeutic properties, including the previously reported and unpublished compounds (the structures of the reported compounds **1**-**72** are in the Supporting Information, Supplementary Table S4). To determine the high and low boundaries for fluorescent intensities, we included negative controls (Mock-membranes) and positive controls (MAGL membrane fractions without compounds) on each 96-well plate. All compounds were tested at 100 µM final concentration, and the activity of MAGL was determined from the slope in the linear region of 5–15 min. In addition,, KML29, a known irreversible MAGL inhibitor, was used in the experiments to verify the results. In the end, the screening of the chemical library identified 26 compounds with greater than 50% inhibition of MAGL (Figure 3A). Among them, eight compounds showing >70% inhibition of MAGL activity were selected for a full determination of dose-response curves based on their potency at 100 µM (indicated with red in Figure 3A). Furthermore, examination of the inhibitory potency demonstrated that four compounds (**9**, **23**, **82,** and **93**) were able to dose-dependently reduce MAGL activity with pIC_50_ values of 4.8 ± 0.1, 4.9 ± 0.1, 5.3 ± 0.1, and 5.8 ± 0.1, respectively (Figures 3B–D; Table 1). The chemical structures of the commercially available compounds **9** and **23** are shown in Figure 3.

**TABLE 1:** Inhibition of recombinant human MAGL, ABHD6, and ABHD12 was determined by indicated assays. Data of pIC_50_ represent average values ±SEM, n = 4 per group. Inhibitory activities of the compounds against ABHD6 and ABHD12 were determined at 100 µM.

Entry	MAGL inhibition (pIC_50_)	ABHD6 inhibition (%)	ABHD12 inhibition (%)
9	4.8 ± 0.1	11%	37%
23	4.9 ± 0.1	37%	18%
82	5.3 ± 0.1	69%	—
83	4.3 ± 0.1	2%	1%
89	4.2 ± 0.1	74%	—
93	5.8 ± 0.1	13%	10%
103	4.3 ± 0.1	—	—
130	4.2 ± 0.1	91%	64%

Next, we evaluated the selectivity of the compounds against MAGL over ABHD6 and ABHD12. For ABHD6, we used a similar assay protocol as MAGL and assessed the inhibitory activity of all the compounds at 100 µM final concentration. The examination revealed that nine compounds demonstrated higher than 50% inhibition towards ABHD6, whereas the remaining compounds showed minimal activity against ABHD6 (Supplementary Figure S6). In analysis of the results from MAGL inhibition vs. ABHD6 inhibition, five compounds (**9**, **23**, **83**, **93,** and **103**) were identified with significant MAGL inhibition (>80% inhibition), but low potency against ABHD6 (<50% inhibition) (Figure 3E), suggesting these compounds are selective MAGL inhibitors over ABHD6 (Table 1). For ABHD12, competitive ABPP was alternatively used to evaluate the inhibitory properties of the compound library (Figure 3G). Briefly, the compounds were screened at a single concentration of 100 µM for the inhibition of human ABHD12 labeling by a fluorophosphonate (FP)-based probe (100 nM FP-TAMRA) (Figure 3G). The percentage of inhibition on ABHD12 activity was calculated and normalized by the samples treated with DMSO (without inhibitors). For the ABPP in-gel analysis, the protein loading was controlled with Coomassie staining gels during the experiments. In the end, the screening results identified six compounds with obvious inhibitory activity towards ABHD12 (>50% inhibition) (Figure 3F, Supplementary Figure S6). Comparing the potency of the compounds against MAGL and ABHD12, compounds **9**, **23**, **82**, **83**, **93,** and **103** were found with neglectable potency towards ABHD12, but significant MAGL inhibition (>80% inhibition).

### Activity and selectivity profile on endogenous MAGL in mouse brain membrane proteome

**FIGURE 4:**
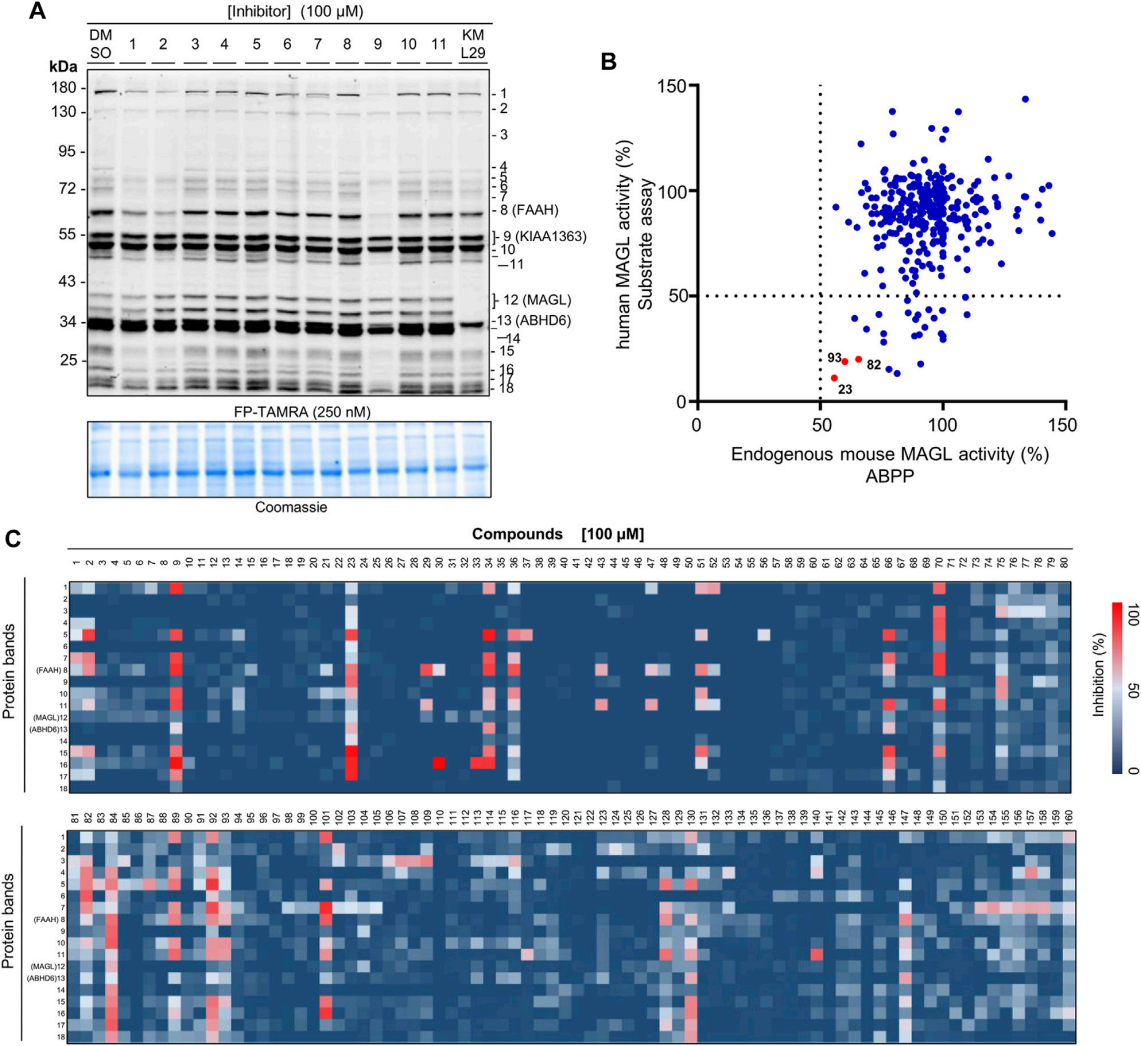
Activity and selectivity profile of the focused compound library using ABPP. **(A)** Representative gel of ABPP assay for determination of the activity and selectivity of compounds **1**-**11**. A known MAGL inhibitor KML29 (10 µM) was used as a control compound. Coomassie staining gel was used as a loading control. **(B)** Analysis of the ABPP assay vs. **AA-HNA** assay against MAGL (100 µM compounds). **(C)** Heatmap overview of the screen of compound selectivity. The inhibition of each protein band was quantified from competitive ABPP gels (e.g., Figure 4A and Supplementary Figures S7, S8). Note that the percentage values were normalized to the intensity of the protein bands from the samples treated with DMSO (vehicle).

To investigate the activity and selectivity of the natural compound library on endogenous MAGL, we applied an ABPP assay with mouse brain membrane proteome. The broad-spectrum serine hydrolase probes such as FP-TAMRA are routinely used in competitive ABPP to evaluate the activity and selectivity of serine hydrolase inhibitors. In our experiments, the compounds at 100 µM were incubated with mouse membrane homogenates for 30 min, subsequently by the addition of FP-TAMRA (250 nM). The results were then analyzed by in-gel-based ABPP and the inhibitory effects on the specific target were calculated and normalized by a DMSO-treated control sample. Of note, Coomassie staining gel was used to correct the protein loading for each sample. Figure 4A showed an example of the result from the gel-based ABPP assay and the percentage of inhibition effect on each protein band is calculated from the obtained gels. Compounds were considered to have significant inhibitory effects when the labeling was reduced by>50%. Analysis of the gel-based ABPP assay vs. previous **AA-HNA**-based substrate assay showed that the compounds were less active in the ABPP assay on endogenous mouse MAGL when compared with the results in the primary screening assay (Figure 4B). As shown in Figure 4C, the informative competitive ABPP results with mouse membrane proteome revealed that compounds **9**, **23**, **34**, **36**, **66**, **70**, **84**, **89**, **92,** and **101** were broad-spectrum inhibitors that cross-reacted with multiple serine hydrolases.

### Antiproliferative screening and potential target identification by ABPP

As reported in the literature, MAGL is a potential target for cancer diseases such as ovarian cancer and colorectal cancer (Nomura et al., 2010; Ye et al., 2011). Thus, we evaluated the overall antiproliferative activity of the compounds against a panel of cancer cell lines, including colon cancer (HT-29), ovarian (OVCAR-3), lung cancer (A549, H1975, HCC827), melanoma (B16-F10), cervical carcinoma (Hela), and epidermal carcinoma (A431), in order to identify the most sensitive cancer cells for these compounds. During the screening, the selected cancer cells were treated with a 20 µM concentration of the compounds for 48 h. MAGL inhibitor KML29 was used as the reference compound. As shown in Supplementary Figure S9, a clear distinction between *in vitro* anti-cancer activity of the compounds in different cancer cells was obtained. Despite most of the compounds showing minimal inhibition of cell viability at concentration of 20 μM, compounds **1**, **23**, **24**, **82**, **93**, **98**, **101**, **102**, **104**, **127,** and **154** produced appreciable inhibition against almost all the tested cell lines. Among them, compounds **64**, **82,** and **93** showed significant potency against the cell viability of A431 with 78, 84, and 80% inhibition, respectively. In particular, **64** selectively inhibited cell proliferation of A431 over other cell lines. Furthermore, compound **95** also showed selective cytotoxic activities against A549 (∼70% inhibition), and was inactive against other cell lines. Besides, compound **9** displayed 54% inhibition of cell viability against B16-F10 and neglectable antiproliferative behavior to other cell lines. To further confirm the anticancer activity, we next measured the IC_50_ values of the selected compounds with significant antiproliferative activity in cancer cells (Table 2). We found compounds **23**, **102**, **104,** and **127** produced significant cytotoxicity towards all eight cancer cell lines with pIC_50_ ≥ 5, suggesting these compounds may have broad-spectrum antitumor activities. The selective MAGL inhibitor KML29 only showed antiproliferative activity against B16-F10 and HT-29 cells, while compounds **23**, **82,** and **93** identified as potential MAGL inhibitors in our study also showed antiproliferative activities against other cancer cells, including A431, H1975, OVCAR-3, and A549. These results implicate that the compounds may have distinct selectivity profiles in different cancer cell lines, thereby contributing to different cytotoxic activities against the cancer cell lines.

**TABLE 2:** Antiproliferation activity of the compounds in a panel of cancer cells.

	Antiproliferation activity (pIC_50_ ± SEM)							
Entry	A431	H1975	B16-F10	OVCAR-3	HT-29	HCC827	Hela	A549
1	<5	<5	5.7 ± 0.2	5.7 ± 0.3	<5	5.1 ± 0.2	5.0 ± 0.2	6.3 ± 0.2
9	<5	<5	<5	<5	<5	<5	<5	<5
17	<5	<5	<5	<5	<5	<5	<5	<5
23	5.1 ± 0.1	5.0 ± 0.1	5.1 ± 0.2	5.3 ± 0.3	5.0 ± 0.1	5.2 ± 0.1	5.1 ± 0.4	5.5 ± 0.3
24	<5	<5	<5	<5	<5	5.0 ± 0.1	<5	<5
64	5.2 ± 0.1	<5	<5	<5	<5	<5	<5	<5
82	5.4 ± 0.1	5.4 ± 0.4	5.6 ± 0.1	5.2 ± 0.1	<5	<5	<5	5.7 ± 0.1
93	5.2 ± 0.6	5.4 ± 0.4	5.8 ± 0.4	5.1 ± 0.1	<5	<5	<5	5.2 ± 0.3
95	<5	<5	<5	<5	<5	<5	<5	<5
98	5.2 ± 0.4	5.6 ± 0.2	5.2 ± 0.1	5.6 ± 0.2	5.3 ± 0.5	<5	5.5 ± 0.3	5.7 ± 0.1
101	5.2 ± 0.3	5.5 ± 0.2	5.0 ± 0.3	5.7 ± 0.1	5.3 ± 0.4	<5	5.5 ± 0.3	5.8 ± 0.2
102	5.4 ± 0.2	5.4 ± 0.5	5.1 ± 0.1	5.6 ± 0.2	5.4 ± 0.1	5.1 ± 0.2	5.3 ± 0.4	5.2 ± 0.2
104	5.7 ± 0.2	5.9 ± 0.2	5.8 ± 0.2	5.5 ± 0.1	5.7 ± 0.2	5.5 ± 0.6	6.2 ± 0.2	5.7 ± 0.3
127	5.6 ± 0.5	5.8 ± 0.2	6.1 ± 0.4	5.9 ± 0.3	5.4 ± 0.4	5.3 ± 0.7	5.7 ± 0.3	5.7 ± 0.1
154	<5	5.4 ± 0.4	5.9 ± 0.3	5.6 ± 0.4	5.9 ± 0.3	5.9 ± 0.2	5.9 ± 0.6	<5
KML29	<5	<5	5.0 ± 0.2	<5	5.0 ± 0.3	<5	<5	<5

**FIGURE 5:**
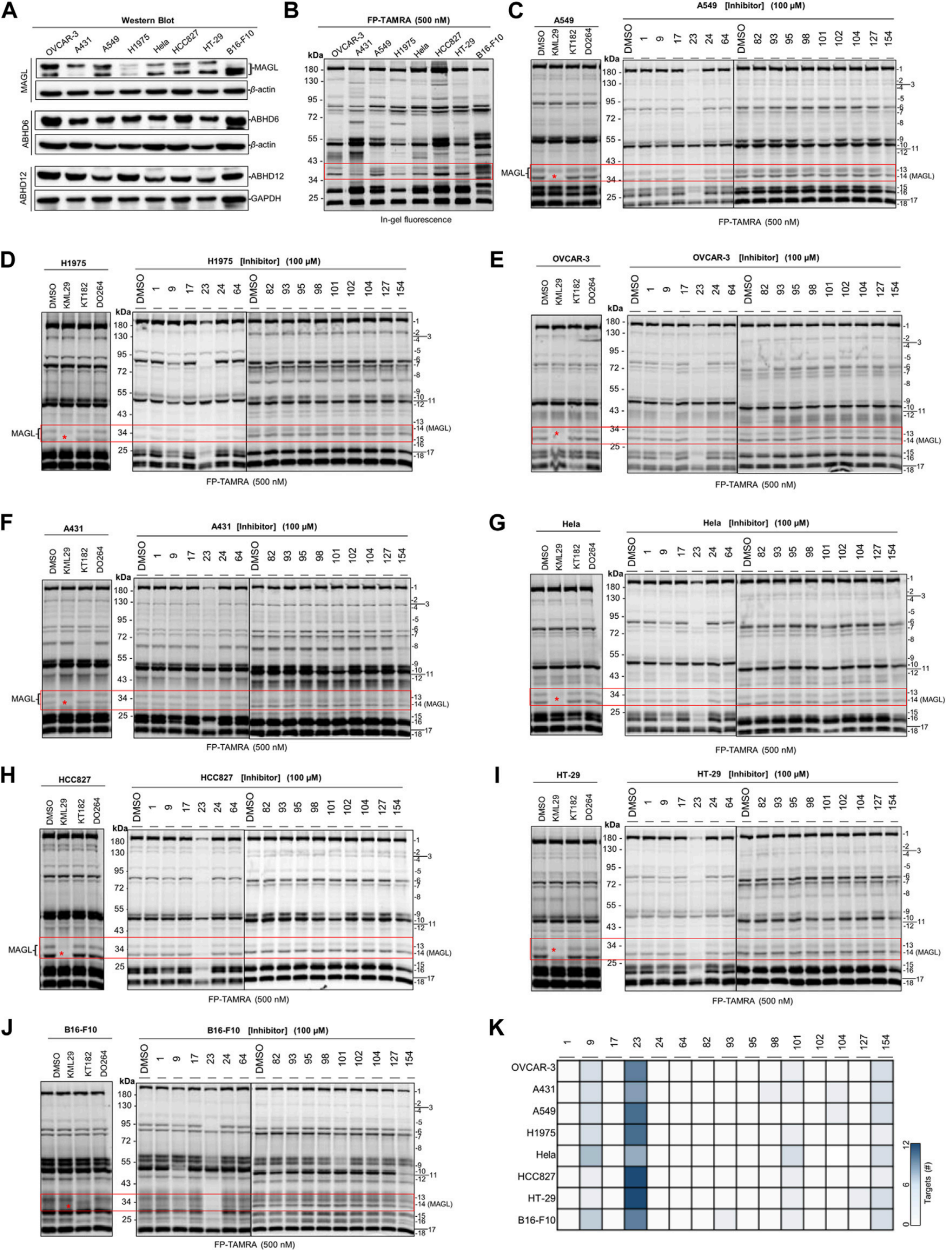
Screening several cancers cells lines to identify the potential target(s) of compounds with antiproliferative activities using competitive ABPP. **(A)** Western blot analysis of the expression of endogenous MAGL, ABHD6, and ABHD12 in the cancer cell lines. **(B)** Comparative ABPP analysis of the potential targets labeled by serine hydrolase probe FP-TAMRA. **(C–J)** Competitive ABPP gel results of compounds **1**, **9**, **17**, **23**, **24**, **64**, **82**, **93**, **95**, **98**, **101**, **102**, **104**, **127,** and **154** show the selectivity profile across serine hydrolases labeled by FP-TAMRA (500 nM). Note that the concentration of KML29, KT182, and DO264 was 10 μM, respectively. **(K)** Heatmap overview of the number of potential targets (identified by the quantification of protein bands in ABPP) for compounds with antiproliferative activities in various cancer cells.

Next, a competitive ABPP assay was applied to screen the cancer cell lines for identifying the potential targets of the compounds with antiproliferative activities. Firstly, we evaluated the expression levels of endogenous MAGL, ABHD6, and ABHD12 in the cancer cells by western blot analysis. Comparative ABPP with serine hydrolase probe FP-TAMRA was also applied to investigate the activity of these enzymes in cancer cells (Figures 5A–J). MAGL inhibitor KML29 (irreversible), ABHD6 inhibitor KT182 (irreversible), and ABHD12 inhibitor DO264 (reversible) were used to confirm the identity of the fluorescent bands labeled by FP-TAMRA. Of note, irreversible inhibitors for ABHD12 are quite limited and DO264 is a reversible competitive inhibitor showing high selectivity and *in vivo* activity towards ABHD12 (Ogasawara et al., 2019). In western blots, high expression levels of MAGL were observed in OVCAR-3 and B16-F10 cells. In addition,, A549, Hela, HCC827, and HT-29 cells were also observed with the expressions of MAGL, whereas A431 and H1975 expressed low levels of MAGL (Figure 5A). However, other endogenous 2-AG hydrolases such as ABHD6 and ABHD12 shared a similar expression pattern in these distinct cell lines (Figure 5A). Comparative ABPP revealed that the activity of MAGL varied in different cancer cell lines (Figure 5B). Furthermore, the fluorescent band of MAGL (∼33 kDa) was competed away by preincubation of KML29, indicating the identity of MAGL in the ABPP gels (Figures 5C–J). The activity of ABHD6 (∼32 kDa) was confirmed in A431 (Figure 5F) and B16-F10 (Figure 5J) cells by competitive ABPP and the labeling of ABHD6 disappeared upon preincubation with KT182.

We next decided to evaluate the activity and selectivity of the compounds (**1**, **9**, **17**, **23**, **24**, **64**, **82**, **93**, **95**, **98**, **101**, **102**, **104**, **127,** and **154**) towards endogenous MAGL in cancer cells. Competitive ABPP with FP-TAMRA was then performed to assess these compounds across a broad panel of serine hydrolases in intact cell lysates. In brief, compounds at 100 µM were incubated for 30 min with cell lysates, followed by a gel-based ABPP analysis using FP-TAMRA (500 nM). As shown in Figure 5, compound **23** completely reduced the labeling of endogenous MAGL in A549 (Figure 5C), H1975 (Figure 5D), OVCAR-3 (Figure 5E), Hela (Figure 5G), HCC827 (Figure 5H), HT-29 (Figure 5I), and B16-F10 (Figure 5J) cells. Furthermore, the in-gel analysis of the ABPP assay clearly indicated that **23** was a broad-spectrum inhibitor by targeting a number of serine hydrolases in cancer cells (e.g., HT-29 and HCC827) (Figure 5K).

### Interaction with the catalytic site of human MAGL: Modeling studies

**FIGURE 6:**
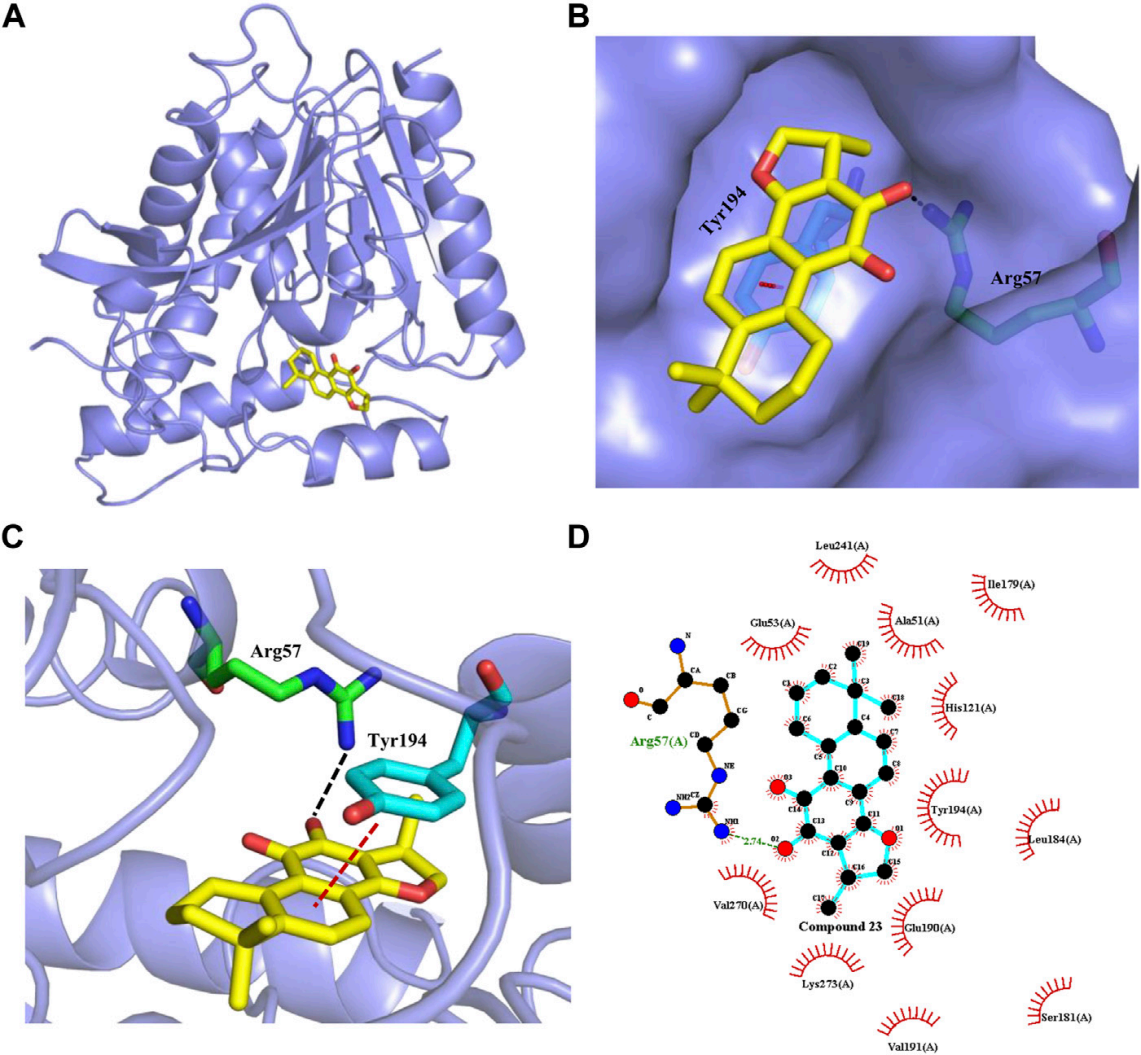
Interaction of cryptotanshinone (**23**, yellow sticks) with human MAGL. **(A)** Overview of molecule docking of compound **23** into human MAGL (PDB code: 5ZUN, light purple ribbon representation). **(B)** Surface rendering model showing the disposition of compound **23** (yellow sticks) in a hydrophobic binding pocket of MAGL (light purple). **(C)** Compound **23** shows the critical interactions with Arg57 and Tyr194. The MAGL domain is shown in ribbon representation. Compound **23** and the residues involved in ligand binding are represented with sticks. The Black dashed line represents a hydrogen bond interaction, while the red dashed line represents a π-π interaction. **(D)** 2D interaction diagram between compound **23** and MAGL. The ligands and protein side chains are shown in ball-and-stick representations with the ligand bonds colored in sky-blue. The hydrogen bond is shown as green dotted lines, while the spoked arcs represent protein residues making nonbonded contacts with compound **23**.

Finally, to explore the potential binding model of cryptotanshinone (**23**) and human MAGL. We performed a molecular docking study of **23** with human MAGL (5ZUN) to provide an insight into any potential interactions. After extracting compound **3i** (1-(3-Phenoxyphenyl)-4-[4-(1,3-thiazol-2-ylcarbonyl)-piperazin-1-yl]pyrrolidin-2-one) from the inhibitor-protein complex, **23** was flexibly docked into MAGL. As presented in Figure 6, **23** could occupy the binding pocket of MAGL. The carbonyl group at the 11-position of **23** could form a direct hydrogen bond with the side chain of Arg57 (Figure 6C). Furthermore, the phenyl moiety of **23** initiated a π-π stacking interaction with Tyr194 (Figure 6C). The dimethylcyclohexane group settled in a hydrophobic pocket including Leu241 and Ala51 residues facilitated by hydrophobic interactions (Figure 6D). In addition, the methyl-2,3-dihydrofuran unit on the other side of **23** was observed to be embedded in the hydrophobic pocket constituted by Val270, Lys273, Val191, and Glu190.

## Discussion

Monoacylglycerol lipases as a serine hydrolase play a crucial role to catalyze the hydrolysis of monoacylglycerol lipids, particularly the endogenous cannabinoid 2-AG in the brain. Selective MAGL inhibitors have been considered as important agents in many therapeutic fields, including anti-nociceptive, anti-inflammatory, and anti-cancer. Despite a number of MAGL inhibitors that have been reported, inhibitors with new chemotypes are still required. In this study, on the basis of the catalytic mechanism of 2-AG hydrolases, we synthesized a new fluorogenic substrate **AA-HNA** and developed an **AA-HNA**-based fluorescence assay. By using human recombinant MAGL, we demonstrate that **AA-HNA** displays a turn-on mechanism when in the presence of 2-AG hydrolases and serves as a fluorogenic substrate for 2-AG hydrolases. To exclude the background fluorescence, we also prepared Mock-membranes (without target enzyme overexpression), which may contain certain components that might cause background fluorescence in the assay. In the end, a single-step enzymatic assay with fluorogenic substrate **AA-HNA** was set up and optimized in our study.

Activity-based protein profiling (ABPP) is a highly useful chemical biological technique to assess the activity and selectivity of inhibitors in a complex native system (e.g., tissue homogenates or intact cell lysates). Yet, to the best of our knowledge, there is a limited description of the combination of ABPP with high-throughput screening assays to evaluate inhibitor activity and selectivity in the earliest stage of lead identification. We, therefore, combined ABPP and our substrate-based fluorescent assay to identify MAGL inhibitors with new chemotypes. In this study, a focused library containing 320 natural organic compounds was successfully screened using an **AA-HNA**-based fluorogenic assay with recombinant human MAGL, and ABPP was combined as an orthogonal method to confirm the inhibitory activity against MAGL in primary screening. The results demonstrate that four compounds (**9**, **23**, **82,** and **93**) were able to dose-dependently reduce MAGL activity. Among them, compound **9**, known as Apigenin (4’,5,7-trihydroxyflavone), is a common flavonoid that widely exists in plants. Apigenin has multiple biological activities, including anti-inflammatory, antioxidant, antibacterial, and antiviral activities (Kumar and Pandey, 2013; Kiraly et al., 2016; Salehi et al., 2019; Tian et al., 2021). Apigenin has also been demonstrated with the suppression of prostaglandins through COX-2 in both microglial and macrophage mouse cells (Kiraly et al., 2016). Moreover, Apigenin has recently been observed to suppress various human cancers *in vitro* and *in vivo* (Choi and Kim, 2009; Sharma et al., 2019; Imran et al., 2020). A quinoid diterpene **23**, named cryptotanshinone, is extracted from the root of the medicinal plant *Salvia miltiorrhiza* and has been reported to exert a diverse range of pharmacological effects such as neuroprotective, anti-fibrosis, anti-inflammatory, and anti-cancer activities (Yang et al., 2018; Wu et al., 2020). The anti-cancer activity of cryptotanshinone (**23**) has been found to be associated with the inhibition of STAT3 phosphorylation (Yang et al., 2018). Compounds **82** and **93**, belonging to *β*-carbolines, are cis- (**82**) and trans-isomers (**93**), have been firstly purified from *Codonopsis pilosula* by our collaborator. Their chemical structures and biological activities of them will be published elsewhere in detail. The selectivity of the compounds towards ABHD6 and ABHD12 was evaluated specifically, both ABPP and substrate assays suggest that compounds **9**, **23,** and **93** are potential inhibitors targeting MAGL, but selective over ABHD6 and ABHD12.

Next, ABPP was applied to investigate the activity and selectivity of the natural compound library on endogenous MAGL in mouse brain membrane proteome. Analysis of the ABPP assay vs. **AA-HNA**-based substrate assay showed that the compounds were less active in the ABPP assay on endogenous mouse MAGL when compared with the results in the substrate assay. The discrepancy in potency between the ABPP assay and the fluorogenic substrate assay might due to the different species of MAGL (endogenous mouse MAGL was used in ABPP, while recombinant human MAGL was used in the substrate assay). Nevertheless, compounds **23**, **82,** and **93** demonstrated significant potency towards recombinant human MAGL and maintained a certain activity against endogenous mouse MAGL in ABPP assay (∼50%–60% inhibition), indicating the cross-species inhibition of these compounds against MAGL. In addition, competitive ABPP enables determination of the selectivity profile over a panel of serine hydrolases in a single experiment, including FAAH and ABHD6. The informative competitive ABPP results with mouse membrane proteome revealed broad-spectrum inhibitors (e.g., **9**, **23**, **34**, **36**, **66**, **70**, **84**, **89**, **92,** and **101**) towards serine hydrolases.

MAGL is a potential target for cancer diseases such as ovarian cancer and colorectal cancer, we, therefore, evaluated the antiproliferative activity of these compounds and applied ABPP to identify the potential targets. To identify the most sensitive cancer cells, we selected distinct cancer cells, including colon cancer (HT-29), ovarian (OVCAR-3), lung cancer (A549, H1975, HCC827), melanoma (B16-F10), cervical carcinoma (Hela,) and epidermal carcinoma (A431). According to the results, we found that four compounds (**23**, **102**, **104,** and **127**) produced significant inhibition against all eight cancer cell lines with pIC_50_ ≥ 5, suggesting these compounds may have broad-spectrum antitumor activities. Moreover, compounds **23**, **82,** and **93**, identified as potential MAGL inhibitors by both ABPP and fluorogenic substrate assay, also showed antiproliferative activities against cancer cells, including A431, H1975, B16-F10, OVCAR-3, and A549. Notably, the selective MAGL inhibitor KML29 only showed antiproliferative activity against B16-F10 and HT-29 cells. These implicated compounds **23**, **82,** and **93** may have other potential off-targets, thereby contributing to the cytotoxic activities against other cancer cell lines (e.g., A431, H1975, OVCAR-3, and A549).

Comparative ABPP with serine hydrolase probe FP-TAMRA revealed that the activity of MAGL was found in most of the cells, but varied in different cancer cell lines, whereas the activity of ABHD6 was only found in A431 and B16-F10. No obvious ABHD12 activity was found in any of the cell lines using ABPP with probe FP-TAMRA. It might be due to the low sensitivity of FP-TAMRA for ABHD12 in the whole cell lysate proteome when compared with ABHD6 and MAGL. Next, competitive ABPP with FP-TAMRA was then applied to assess the activity and selectivity of the compounds towards endogenous MAGL in these cancer cells. No potential target was found for compounds **1**, **17**, **24**, **64**, **82,** and **95** in the cancer cells by ABPP in-gel analysis. Surprisingly, no obvious inhibition against endogenous MAGL was observed by compounds **82** and **93** in any of the tested cancer cell lines, however, the two compounds were potent MAGL inhibitors identified by both ABPP and fluorogenic substrate assay. In addition, compounds **98**, **101**, **102**, **104**, **127,** and **154** showed significant antiproliferative behavior in cancer cells, but with no significant MAGL inhibition. These compounds also showed neglectable inhibition towards other serine hydrolases, suggesting the existence of potential target(s) that cannot be detected by FP-TAMRA. Notably, the selectivity evaluation in our study was only limited by serine hydrolases labeled by FP-TAMRA. As compounds derived from natural products often have multiple targets, a comprehensive selectivity profile of these compounds is still required in whole protein classes to elucidate the antiproliferative behavior in cancer cells. The study of proteome-wide selectivity for compounds with antiproliferative potency may also have the potential to discover new anticancer targets, as well as anticancer drug candidates.

In summary, we synthesized a new fluorogenic substrate **AA-HNA** and developed an **AA-HNA**-based fluorescence assay to rapidly identify MAGL inhibitors. In combination with ABPP, we screened a focused library containing 320 natural organic compounds with an **AA-HNA**-based substrate assay. Our investigations culminated in the identification of two major compound classes, including quinoid diterpene (**23**, cryptotanshinone) and *β*-carbolines (**82** and **93**, *cis*- and *trans*-isomers), with significant potency towards MAGL and good selectivity over other 2-AG hydrolases (ABHD6 and ABHD12). Furthermore, we also found these compounds showed appreciable antiproliferative activities against several cancer cells, including A431, H1975, B16-F10, OVCAR-3, and A549. Importantly, competitive ABPP results revealed that **23,** but not **82** and **93**, showed nearly complete inhibition against endogenous MAGL in eight cancer cells. In addition, the molecular modeling studies also provide the structural basis for **23** as a potential MAGL inhibitor. Our results demonstrated the potential utility of the fluorogenic substrate assay in combination with ABPP for rapid screening of MAGL inhibitors with therapeutic potential, and the importance of employing ABPP to identify potential targets for compounds with significant biological activities.

## Data Availability

The original contributions presented in the study are included in the article/Supplementary Material; further inquiries can be directed to the corresponding authors.
