# Supplementary material for: Discovering monoacylglycerol lipase inhibitors by a combination of fluorogenic substrate assay and activity-based protein profiling
Source: Front Pharmacol. 2022 Aug 29;13:941522. doi: 10.3389/fphar.2022.941522 (PMC9465256; doi:10.3389/fphar.2022.941522)
Supplement: Supplementary file 1 [file DataSheet1.docx]

Supplementary Material

# Supplementary Data

**NMR (^1^H and ^13^C)**

6-Formylnaphthalen-2-yl (5Z,8Z,11Z,14Z)-icosa-5,8,11,14-tetraenoate (**AA-HNA**)


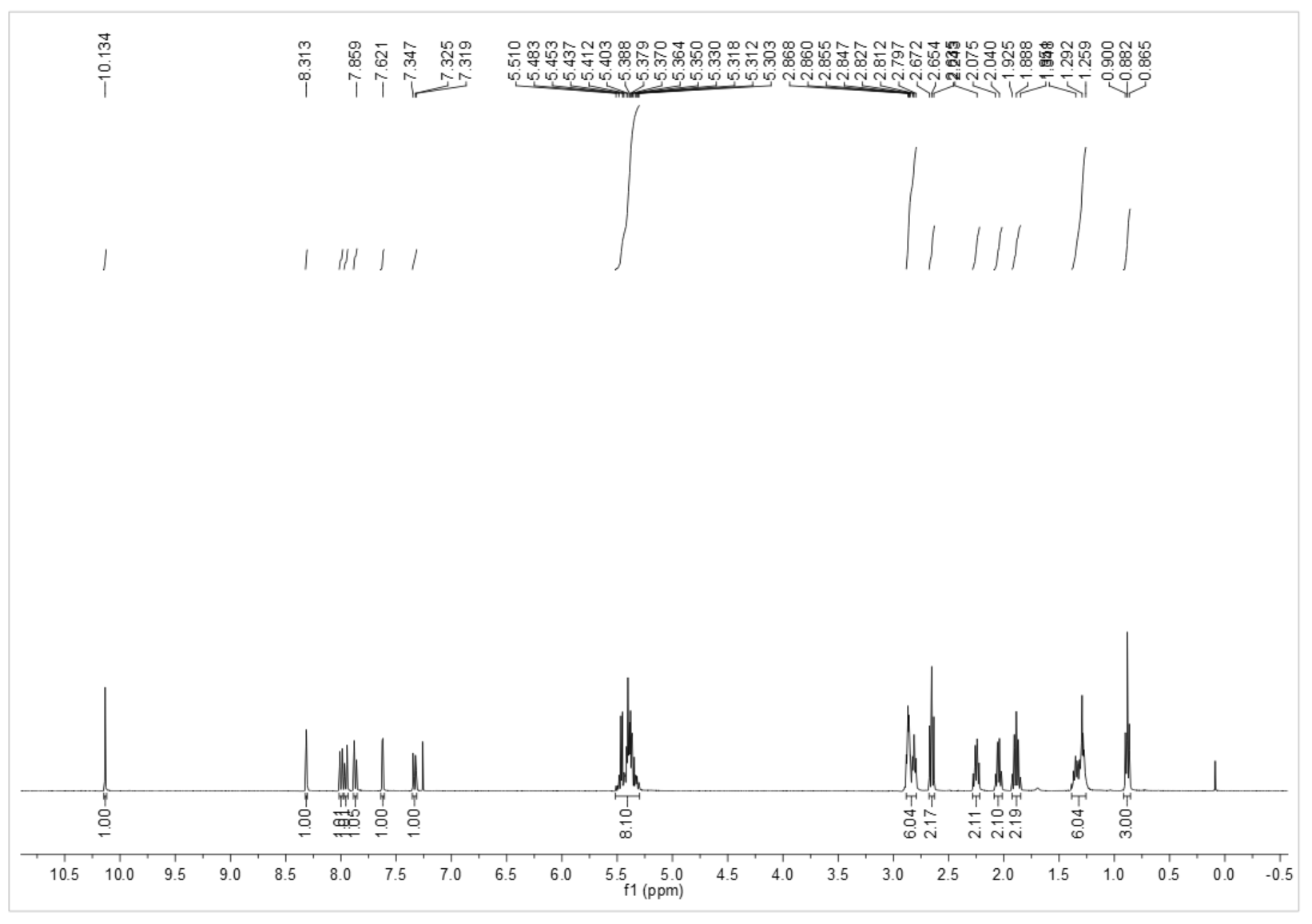


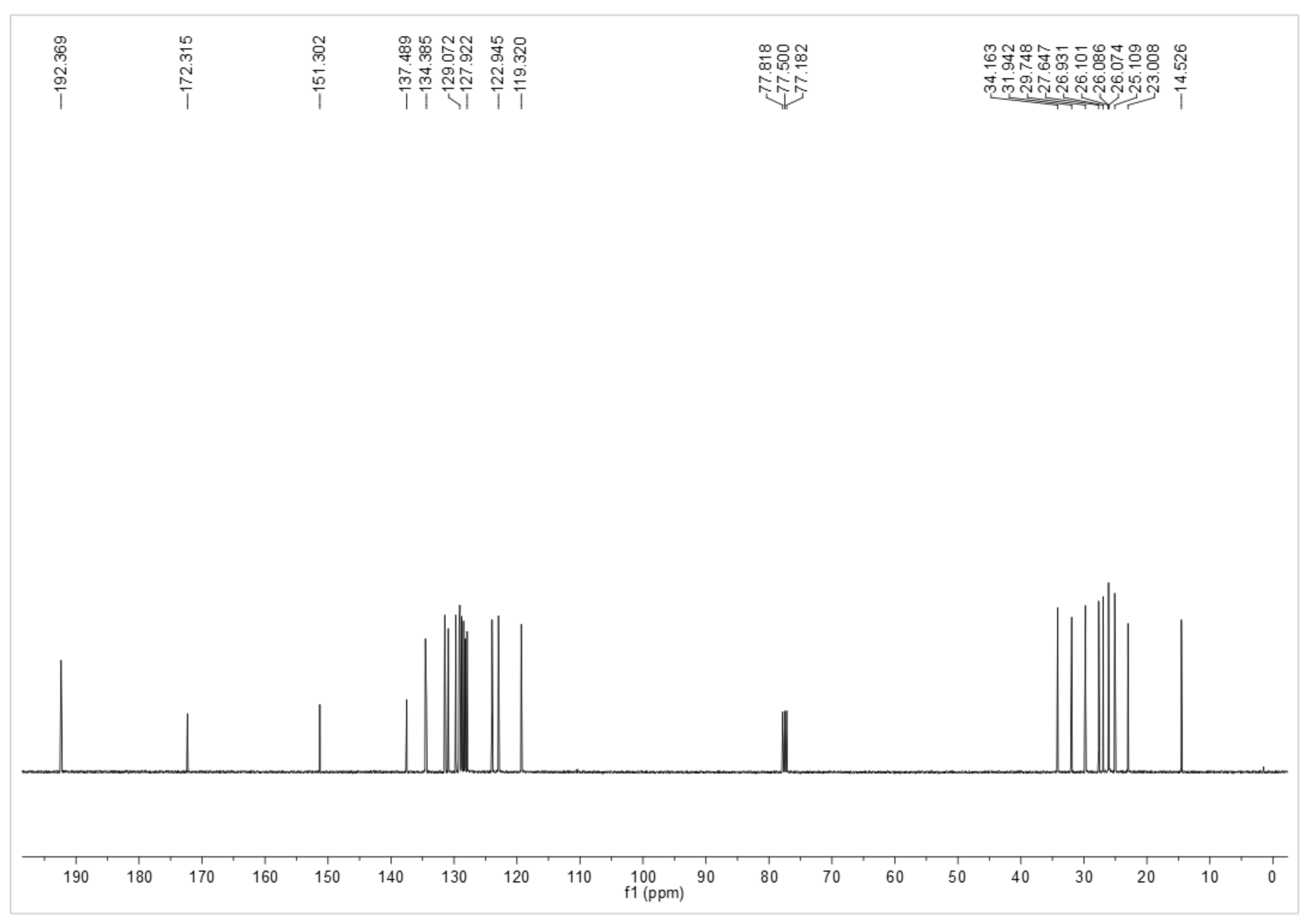


**HRMS**

6-Formylnaphthalen-2-yl (5Z,8Z,11Z,14Z)-icosa-5,8,11,14-tetraenoate (**AA-HNA**)

# Supplementary Figures and Tables

## Supplementary Figures


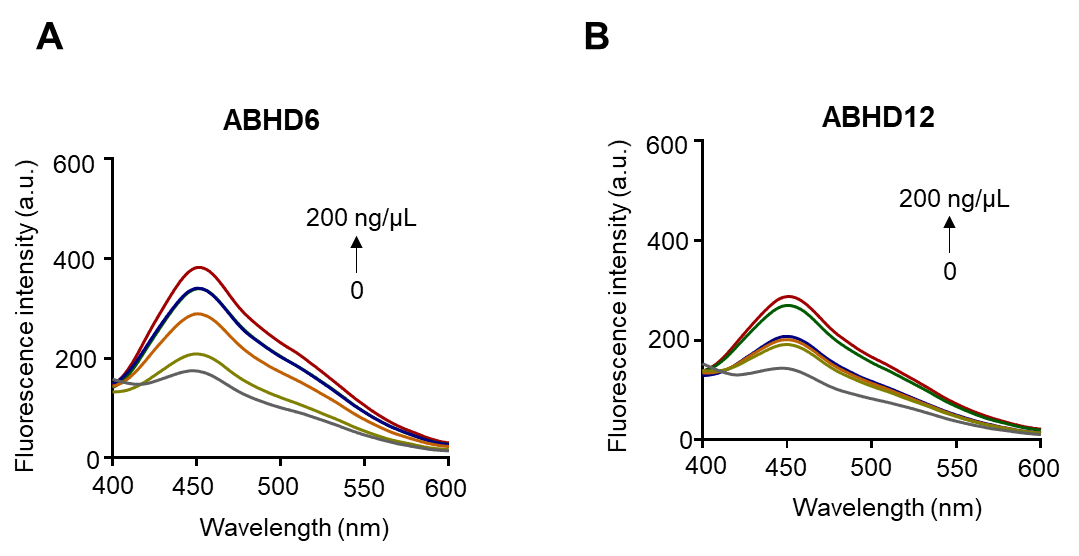


**Supplementary Figure 1.** The concentration-dependent fluorescence spectra response of fluorescence intensity at the emission of 455 nm with the increasing of protein concentration of ABHD6 (A) and ABHD12 (B).


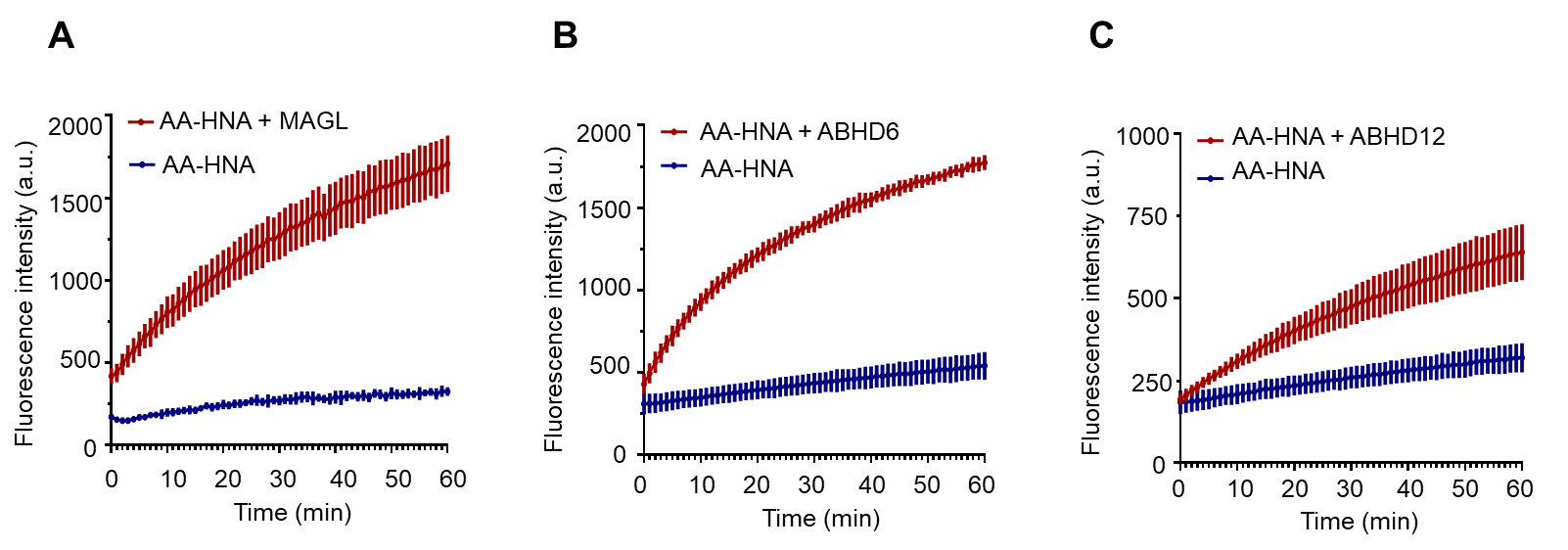


**Supplementary Figure 2.** (A) The time-dependent increase of fluorescence intensity in the presence of MAGL and fluorogenic substrate AA-HNA. (B) The time-dependent increase of fluorescence intensity in the presence of ABHD6 and fluorogenic substrate AA-HNA. (C) The time-dependent increase of fluorescence intensity in the presence of ABHD12 and fluorogenic substrate AA-HNA. Note that the excitation and emission was 330 nm and 455 nm, respectively.


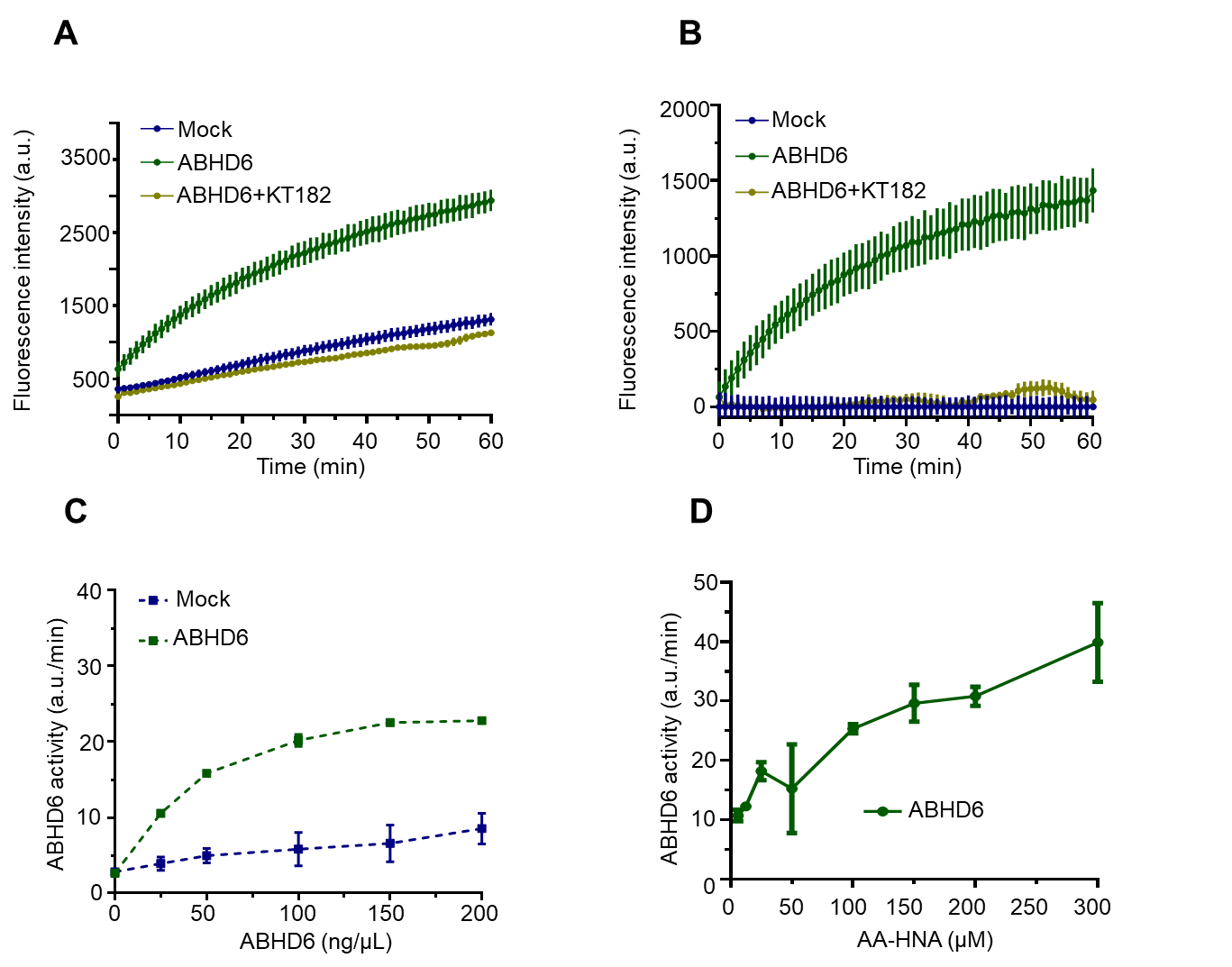


**Supplementary Figure 3.** Setup and optimization of **AA-HNA**-based activity assay for ABHD6. (**A**) Time course of **AA-HNA** hydrolysis by ABHD6, resulting in an increase of fluorescence over time. Preincubation with ABHD6 inhibitor KT182 (10 µM) resulted in reduction of the fluorescent signal. Membrane proteins from Mock-transfected cells served as a negative control. (**B**) Time course of 2-AG hydrolysis by ABHD6, corrected for background fluorescence of the Mock-transfected negative control. (**C**) Optimization of protein concentration for the ABHD6 activity assay. ABHD6 activity was monitored in time with various concentrations of ABHD6. (**D**) Optimization of **AA-HNA** concentration for the ABHD6 activity assay.


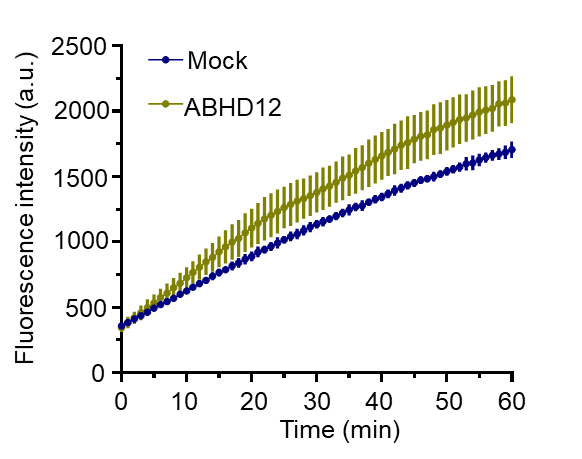


**Supplementary Figure 4.** Time course of AA-HNA hydrolysis by ABHD12 and Mock-membrane, resulting in an increase of fluorescence over time.


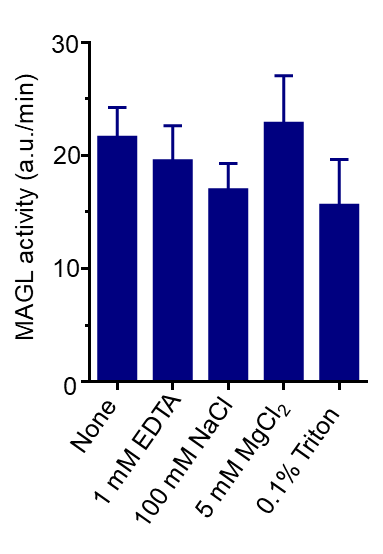


**Supplementary Figure 5.** Optimization of **AA-HNA**-based activity assay for MAGL.


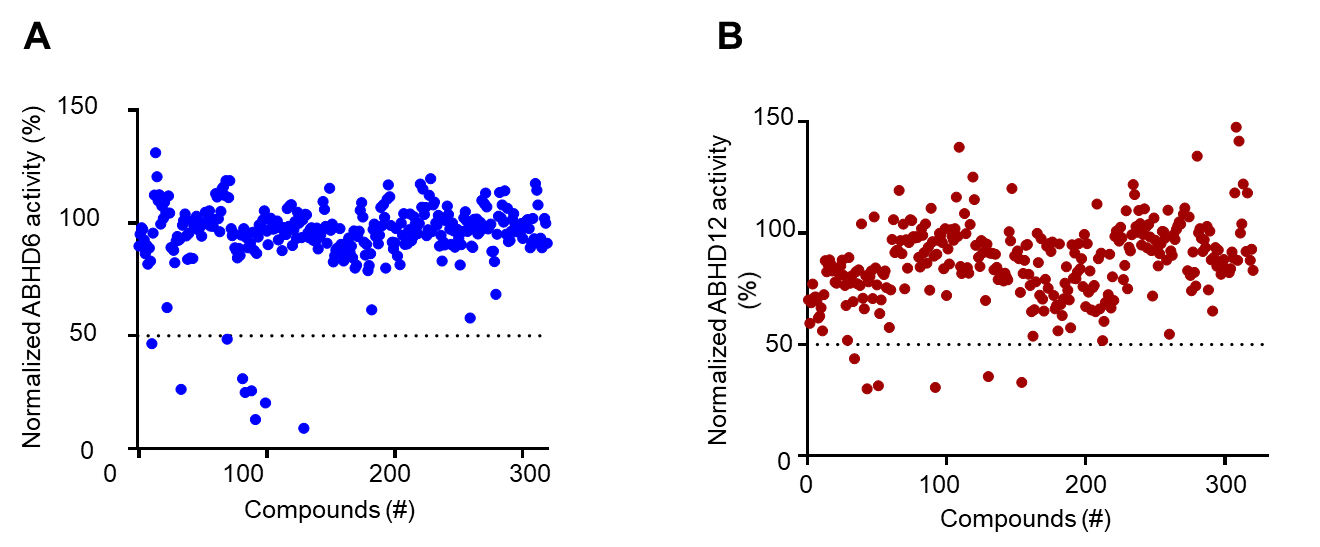


**Supplementary Figure 6.** Screening results of the ~320 focused library against the activity of ABHD6 (A) and ABHD12 (B).


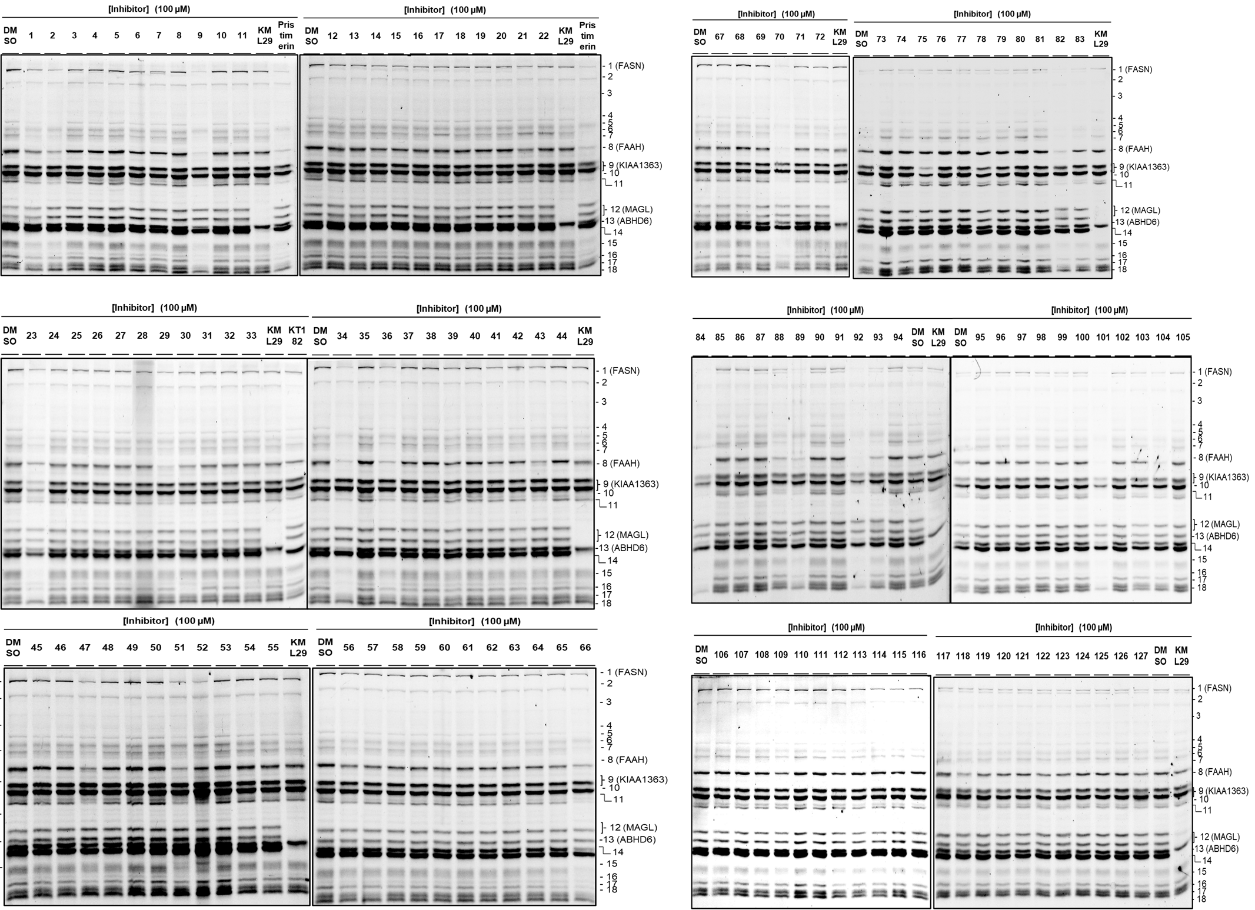


**Supplementary Figure 7.** Representative gels of ABPP assay for determination of the selectivity of compounds **1**-**127**.


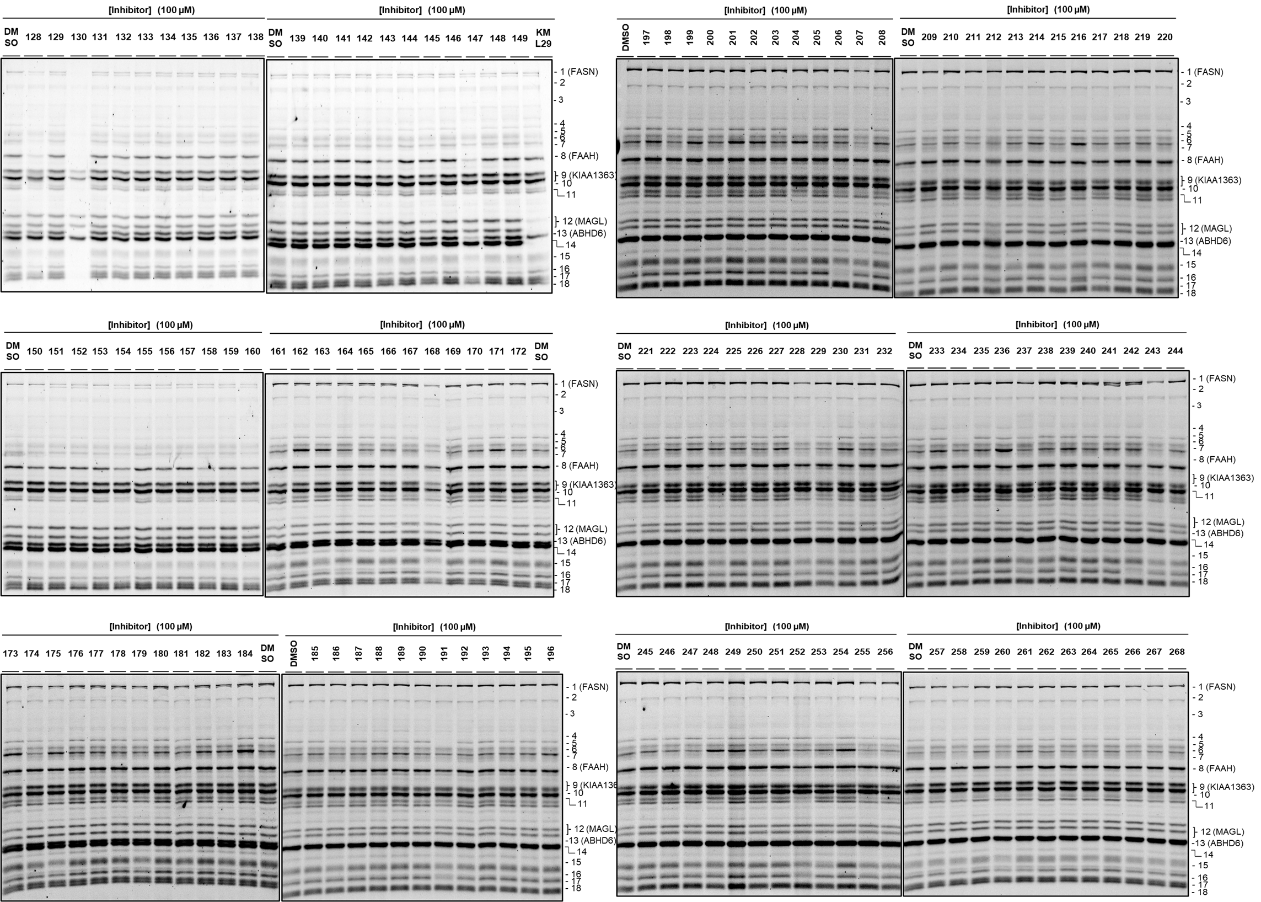

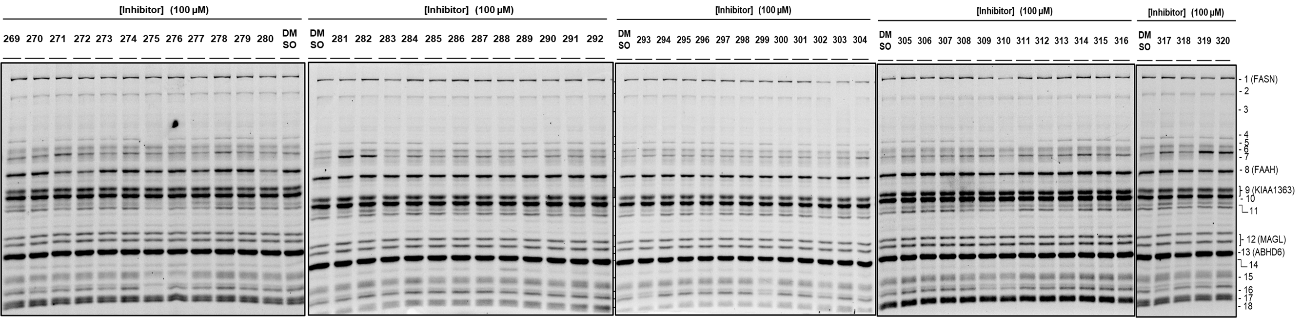


**Supplementary Figure 8.** Representative gels of ABPP assay for determination of the selectivity of compounds **128**-**320**.


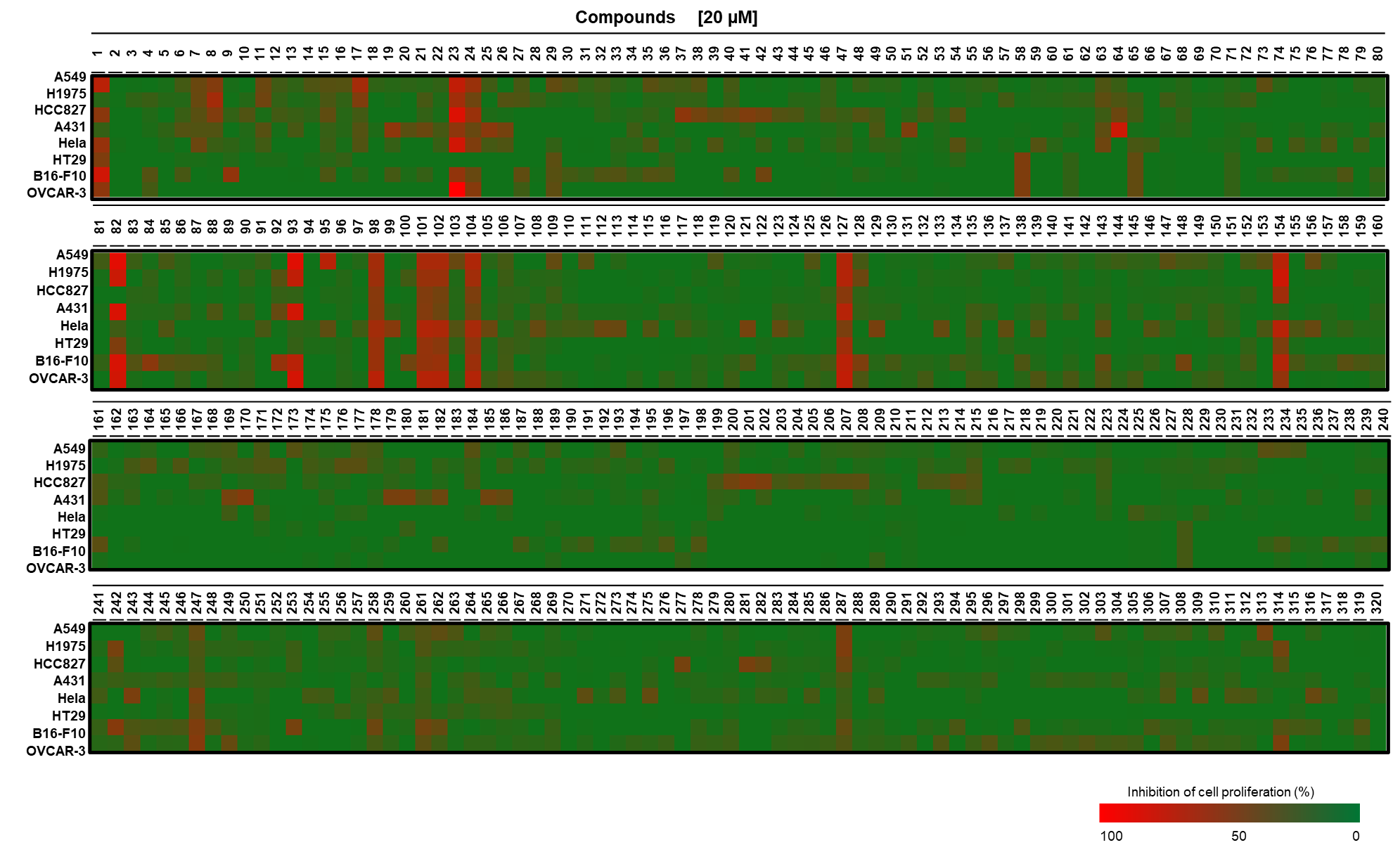


**Supplementary Figure 9.** Heatmap overview of the antiproliferative screening of the natural organic compound library against a panel of cancer cell lines, including lung cancer (A549, H1975, HCC827), epidermal carcinoma (A431), cervical carcinoma (Hela), colon cancer (HT-29), melanoma (B16-F10) and ovarian (OVCAR-3).


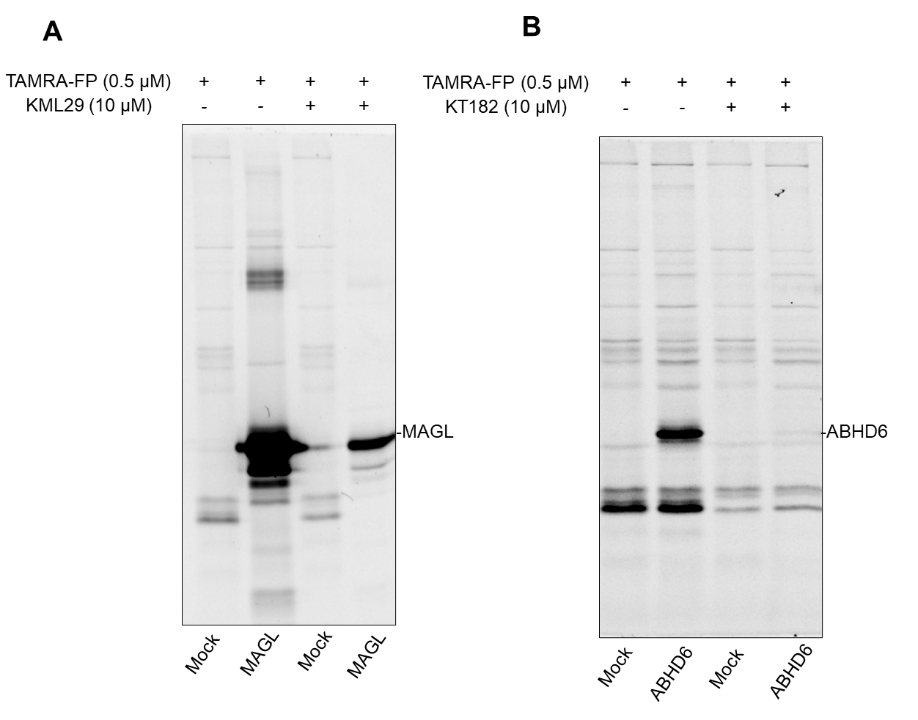


**Supplementary Figure 10.** ABPP assay demonstrates HEK293T cells overexpressing human MAGL and ABHD6. TAMRA-FP (0.5 µM) was used as an activity-based probe to label recombinant MAGL or ABHD6, and the labelling can be blocked by pre-treated with inhibitors KT109 or KT182.

## Supplementary Tables

**Supplementary Table 1.** Inhibitory values for 320 natural organic compounds at 100 µM against MAGL determined by **AA-HNA** substrate assay.

| Entry | MAGL Inhibition (%) | Entry | MAGL Inhibition (%) | Entry | MAGL Inhibition (%) | Entry | MAGL Inhibition (%) |
| --- | --- | --- | --- | --- | --- | --- | --- |
| **1** | 31.7 | **81** | 77.6 | **161** | 88.3 | **241** | 86.8 |
| **2** | 38.9 | **82** | 20.1 | **162** | 90.9 | **242** | 93.9 |
| **3** | 85.6 | **83** | 15.3 | **163** | 92.6 | **243** | 92.2 |
| **4** | 85.4 | **84** | 34.3 | **164** | 82.4 | **244** | 60.8 |
| **5** | 90.1 | **85** | 82 | **165** | 89.7 | **245** | 86.3 |
| **6** | 31.1 | **86** | 77.7 | **166** | 77.1 | **246** | 76.3 |
| **7** | 75.9 | **87** | 77.7 | **167** | 66.8 | **247** | 74.9 |
| **8** | 83.5 | **88** | 31 | **168** | 88.1 | **248** | 74 |
| **9** | 13.3 | **89** | 29.4 | **169** | 89.7 | **249** | 65.3 |
| **10** | 84.2 | **90** | 89.2 | **170** | 93.4 | **250** | 86 |
| **11** | 105.7 | **91** | 83.9 | **171** | 126.9 | **251** | 65 |
| **12** | 86 | **92** | 39.4 | **172** | 72.5 | **252** | 87.2 |
| **13** | 89.9 | **93** | 18.9 | **173** | 96.6 | **253** | 81.8 |
| **14** | 64.3 | **94** | 79.3 | **174** | 92.4 | **254** | 87.4 |
| **15** | 80.3 | **95** | 88.9 | **175** | 99.4 | **255** | 88.2 |
| **16** | 124.4 | **96** | 79.3 | **176** | 128.9 | **256** | 92.7 |
| **17** | 82.8 | **97** | 70.9 | **177** | 88.7 | **257** | 58.5 |
| **18** | 97.8 | **98** | 78.6 | **178** | 102.7 | **258** | 81.9 |
| **19** | 90.1 | **99** | 89.3 | **179** | 92.4 | **259** | 87.9 |
| **20** | 95.2 | **100** | 39.6 | **180** | 102 | **260** | 89.5 |
| **21** | 59.3 | **101** | 84 | **181** | 86.3 | **261** | 86.6 |
| **22** | 93.2 | **102** | 87.2 | **182** | 97.3 | **262** | 88 |
| **23** | 11.2 | **103** | 17.8 | **183** | 92.3 | **263** | 90.1 |
| **24** | 99.1 | **104** | 74.9 | **184** | 101 | **264** | 85.7 |
| **25** | 99 | **105** | 88.5 | **185** | 96.9 | **265** | 91.1 |
| **26** | 92.5 | **106** | 87.7 | **186** | 93.2 | **266** | 83.8 |
| **27** | 97.6 | **107** | 84 | **187** | 97 | **267** | 81.7 |
| **28** | 63.2 | **108** | 93.4 | **188** | 81.1 | **268** | 89 |
| **29** | 76.2 | **109** | 82 | **189** | 90.2 | **269** | 73.2 |
| **30** | 95.9 | **110** | 68.8 | **190** | 93.9 | **270** | 88.4 |
| **31** | 102.7 | **111** | 106.7 | **191** | 114.9 | **271** | 98 |
| **32** | 104 | **112** | 51.6 | **192** | 106.9 | **272** | 86.1 |
| **33** | 99.5 | **113** | 79.3 | **193** | 102.6 | **273** | 101.7 |
| **34** | 35.2 | **114** | 93.9 | **194** | 97.2 | **274** | 104.4 |
| **35** | 97.3 | **115** | 100.3 | **195** | 96 | **275** | 103.2 |
| **36** | 47.9 | **116** | 101.4 | **196** | 75 | **276** | 99.1 |
| **37** | 90.9 | **117** | 104.3 | **197** | 95.7 | **277** | 98.3 |
| **38** | 91.4 | **118** | 100.7 | **198** | 91.3 | **278** | 89.3 |
| **39** | 111.3 | **119** | 95.3 | **199** | 101.6 | **279** | 91.3 |
| **40** | 97 | **120** | 86.5 | **200** | 99.8 | **280** | 79.8 |
| **41** | 84 | **121** | 102.8 | **201** | 91.6 | **281** | 108.8 |
| **42** | 70.6 | **122** | 94 | **202** | 86.6 | **282** | 105.3 |
| **43** | 32.2 | **123** | 62.3 | **203** | 76.8 | **283** | 98.8 |
| **44** | 90.5 | **124** | 91.6 | **204** | 92.6 | **284** | 104.4 |
| **45** | 81.1 | **125** | 92.1 | **205** | 82 | **285** | 103.5 |
| **46** | 90.6 | **126** | 97.1 | **206** | 91.9 | **286** | 101.4 |
| **47** | 43.2 | **127** | 62.2 | **207** | 84.9 | **287** | 86.5 |
| **48** | 72.2 | **128** | 32.2 | **208** | 93.2 | **288** | 83.6 |
| **49** | 85.4 | **129** | 109.9 | **209** | 94.2 | **289** | 94.2 |
| **50** | 62.5 | **130** | 28.2 | **210** | 94.7 | **290** | 67.8 |
| **51** | 49.4 | **131** | 103.5 | **211** | 85.1 | **291** | 100.6 |
| **52** | 41.2 | **132** | 105.5 | **212** | 96 | **292** | 106.1 |
| **53** | 93.1 | **133** | 97.9 | **213** | 91.8 | **293** | 89.7 |
| **54** | 143.4 | **134** | 105.6 | **214** | 137.4 | **294** | 107.3 |
| **55** | 104.4 | **135** | 105.6 | **215** | 107.4 | **295** | 122.2 |
| **56** | 87.4 | **136** | 106.9 | **216** | 99.3 | **296** | 98.3 |
| **57** | 86.8 | **137** | 106.1 | **217** | 97.7 | **297** | 106.2 |
| **58** | 83.4 | **138** | 88.3 | **218** | 101.8 | **298** | 96.8 |
| **59** | 129.6 | **139** | 108.6 | **219** | 137.6 | **299** | 106.9 |
| **60** | 59.6 | **140** | 114.6 | **220** | 99.2 | **300** | 94.7 |
| **61** | 101.1 | **141** | 109.1 | **221** | 93.5 | **301** | 104.2 |
| **62** | 94.5 | **142** | 96.7 | **222** | 91.6 | **302** | 100.7 |
| **63** | 99.9 | **143** | 76.4 | **223** | 96.6 | **303** | 75.3 |
| **64** | 101.7 | **144** | 74.8 | **224** | 89.1 | **304** | 78.1 |
| **65** | 87.5 | **145** | 103 | **225** | 96.1 | **305** | 105.8 |
| **66** | 41.3 | **146** | 82.3 | **226** | 78.1 | **306** | 102.4 |
| **67** | 75.9 | **147** | 43.5 | **227** | 86.5 | **307** | 95.2 |
| **68** | 99.7 | **148** | 56.1 | **228** | 95.7 | **308** | 79.7 |
| **69** | 94.8 | **149** | 79.7 | **229** | 91.3 | **309** | 81.4 |
| **70** | 42 | **150** | 54.8 | **230** | 84.1 | **310** | 96.2 |
| **71** | 99.2 | **151** | 84.6 | **231** | 92.1 | **311** | 100.8 |
| **72** | 75.9 | **152** | 98.7 | **232** | 88.5 | **312** | 78.5 |
| **73** | 89.7 | **153** | 85.2 | **233** | 92.3 | **313** | 98.8 |
| **74** | 101.1 | **154** | 41.1 | **234** | 94 | **314** | 91.6 |
| **75** | 66.3 | **155** | 62.4 | **235** | 89.8 | **315** | 81.7 |
| **76** | 84.4 | **156** | 80.1 | **236** | 90.7 | **316** | 99.2 |
| **77** | 70.2 | **157** | 90.1 | **237** | 85.1 | **317** | 93.2 |
| **78** | 63.1 | **158** | 74.9 | **238** | 85.4 | **318** | 96.5 |
| **79** | 71.2 | **159** | 98 | **239** | 82.5 | **319** | 85 |
| **80** | 62 | **160** | 102.3 | **240** | 86 | **320** | 112.9 |

**Supplementary Table 2.** Inhibitory values for 320 natural organic compounds at 100 µM against ABHD6 determined by **AA-HNA** substrate assay.

| Entry | ABHD6 Inhibition (%) | Entry | ABHD6 Inhibition (%) | Entry | ABHD6 Inhibition (%) | Entry | ABHD6 Inhibition (%) |
| --- | --- | --- | --- | --- | --- | --- | --- |
| **1** | 89.8 | **81** | 94.7 | **161** | 83.2 | **241** | 101 |
| **2** | 95 | **82** | 31 | **162** | 84.3 | **242** | 107.6 |
| **3** | 97.9 | **83** | 97.6 | **163** | 86.8 | **243** | 103.8 |
| **4** | 97.2 | **84** | 24.9 | **164** | 91.7 | **244** | 97.3 |
| **5** | 92.5 | **85** | 93.6 | **165** | 93.4 | **245** | 90.5 |
| **6** | 86.4 | **86** | 89.1 | **166** | 87 | **246** | 100 |
| **7** | 90.2 | **87** | 95.6 | **167** | 89.8 | **247** | 96.5 |
| **8** | 81.8 | **88** | 91.1 | **168** | 84.7 | **248** | 89.3 |
| **9** | 88.9 | **89** | 25.7 | **169** | 96.6 | **249** | 96.2 |
| **10** | 83.2 | **90** | 95.9 | **170** | 80 | **250** | 97.2 |
| **11** | 46.5 | **91** | 87.9 | **171** | 81.3 | **251** | 93.2 |
| **12** | 95.6 | **92** | 12.9 | **172** | 94.1 | **252** | 81.4 |
| **13** | 112.4 | **93** | 86.5 | **173** | 96.4 | **253** | 90.4 |
| **14** | 131.2 | **94** | 93.8 | **174** | 105.7 | **254** | 96.3 |
| **15** | 120.6 | **95** | 99.2 | **175** | 109.1 | **255** | 102.3 |
| **16** | 109.9 | **96** | 97.1 | **176** | 102.7 | **256** | 105 |
| **17** | 112.5 | **97** | 98.2 | **177** | 91.5 | **257** | 101.1 |
| **18** | 99.5 | **98** | 101.4 | **178** | 85.8 | **258** | 96.3 |
| **19** | 107.5 | **99** | 105.4 | **179** | 90.6 | **259** | 98.4 |
| **20** | 102.1 | **100** | 20.3 | **180** | 78.9 | **260** | 57.9 |
| **21** | 109.2 | **101** | 96.4 | **181** | 81.2 | **261** | 89.8 |
| **22** | 104.7 | **102** | 90.4 | **182** | 86.4 | **262** | 89.2 |
| **23** | 62.5 | **103** | 101.5 | **183** | 61.6 | **263** | 101.7 |
| **24** | 112 | **104** | 102.2 | **184** | 97.2 | **264** | 97.3 |
| **25** | 104.4 | **105** | 98 | **185** | 99.6 | **265** | 101.8 |
| **26** | 89.2 | **106** | 97.3 | **186** | 94 | **266** | 95.2 |
| **27** | 88.9 | **107** | 99.2 | **187** | 95.1 | **267** | 95.9 |
| **28** | 87.9 | **108** | 97.8 | **188** | 90.8 | **268** | 100.3 |
| **29** | 82.4 | **109** | 100.9 | **189** | 94.7 | **269** | 110.1 |
| **30** | 92 | **110** | 92.5 | **190** | 106.9 | **270** | 97.6 |
| **31** | 94.1 | **111** | 98 | **191** | 98.8 | **271** | 108.6 |
| **32** | 92.4 | **112** | 98.1 | **192** | 108.2 | **272** | 113.3 |
| **33** | 92.9 | **113** | 87.8 | **193** | 102.6 | **273** | 107.2 |
| **34** | 26.3 | **114** | 98 | **194** | 80 | **274** | 97.4 |
| **35** | 99 | **115** | 95 | **195** | 110.6 | **275** | 98.7 |
| **36** | 101.2 | **116** | 94.9 | **196** | 116.9 | **276** | 99.5 |
| **37** | 104.1 | **117** | 106.4 | **197** | 111.7 | **277** | 87.4 |
| **38** | 94.3 | **118** | 98.6 | **198** | 98.9 | **278** | 87.5 |
| **39** | 83.9 | **119** | 107.7 | **199** | 91.6 | **279** | 82.9 |
| **40** | 95.4 | **120** | 108.2 | **200** | 88.6 | **280** | 68.4 |
| **41** | 84.6 | **121** | 90 | **201** | 91.3 | **281** | 95.1 |
| **42** | 100.9 | **122** | 97 | **202** | 97.5 | **282** | 103.1 |
| **43** | 84.3 | **123** | 100.7 | **203** | 85.4 | **283** | 113.5 |
| **44** | 98.5 | **124** | 93.2 | **204** | 85.3 | **284** | 102.3 |
| **45** | 98.8 | **125** | 104.8 | **205** | 81.5 | **285** | 108.1 |
| **46** | 103 | **126** | 99.9 | **206** | 100 | **286** | 100.4 |
| **47** | 97 | **127** | 103 | **207** | 94.5 | **287** | 114.3 |
| **48** | 100.8 | **128** | 93.6 | **208** | 104.2 | **288** | 95 |
| **49** | 98.3 | **129** | 101.4 | **209** | 90.6 | **289** | 106.5 |
| **50** | 94.2 | **130** | 9.1 | **210** | 91.1 | **290** | 91.8 |
| **51** | 99.4 | **131** | 103.9 | **211** | 90.8 | **291** | 92.3 |
| **52** | 100.1 | **132** | 103.7 | **212** | 97 | **292** | 96.3 |
| **53** | 104.8 | **133** | 95.7 | **213** | 104.1 | **293** | 101.7 |
| **54** | 100.5 | **134** | 93.5 | **214** | 92.2 | **294** | 104.2 |
| **55** | 103.8 | **135** | 97.4 | **215** | 93.5 | **295** | 99.4 |
| **56** | 105.4 | **136** | 97.9 | **216** | 102.8 | **296** | 96.2 |
| **57** | 98.7 | **137** | 92.4 | **217** | 99.6 | **297** | 96.3 |
| **58** | 100.5 | **138** | 95.1 | **218** | 98.3 | **298** | 96 |
| **59** | 98.5 | **139** | 91.9 | **219** | 95.3 | **299** | 95.7 |
| **60** | 101.5 | **140** | 97.8 | **220** | 99.8 | **300** | 99.8 |
| **61** | 113 | **141** | 88.5 | **221** | 117.3 | **301** | 93.2 |
| **62** | 111.5 | **142** | 93.7 | **222** | 107 | **302** | 98.7 |
| **63** | 101.9 | **143** | 94.8 | **223** | 115.1 | **303** | 98.2 |
| **64** | 96.2 | **144** | 97 | **224** | 103.8 | **304** | 101 |
| **65** | 105.2 | **145** | 109.5 | **225** | 106.9 | **305** | 101.5 |
| **66** | 115.2 | **146** | 106.1 | **226** | 94.4 | **306** | 97.5 |
| **67** | 116 | **147** | 98.5 | **227** | 97 | **307** | 89 |
| **68** | 112.3 | **148** | 91.1 | **228** | 112.3 | **308** | 91.4 |
| **69** | 118.8 | **149** | 97.7 | **229** | 119.7 | **309** | 102.1 |
| **70** | 48.6 | **150** | 115.4 | **230** | 96.2 | **310** | 93 |
| **71** | 111.3 | **151** | 96.8 | **231** | 108.1 | **311** | 117.6 |
| **72** | 118.8 | **152** | 99.5 | **232** | 109.3 | **312** | 114.6 |
| **73** | 97.5 | **153** | 82.8 | **233** | 100.1 | **313** | 108.1 |
| **74** | 94.4 | **154** | 85.5 | **234** | 99 | **314** | 90.8 |
| **75** | 95.4 | **155** | 88.1 | **235** | 103.4 | **315** | 93.3 |
| **76** | 89.1 | **156** | 88.9 | **236** | 89.8 | **316** | 89 |
| **77** | 87.8 | **157** | 87.5 | **237** | 94.6 | **317** | 91.4 |
| **78** | 84.6 | **158** | 97.5 | **238** | 83.2 | **318** | 102.1 |
| **79** | 88.8 | **159** | 90.6 | **239** | 91.5 | **319** | 99.9 |
| **80** | 85.7 | **160** | 88.1 | **240** | 90.3 | **320** | 91 |

**Supplementary Table 3.** Inhibitory values for 320 natural organic compounds at 100 µM against ABHD12 determined by competitive activity-based protein profiling (ABPP) with probe FP-TAMRA (250 nM).

| Entry | ABHD12 Inhibition (%) | Entry | ABHD12 Inhibition (%) | Entry | ABHD12 Inhibition (%) | Entry | ABHD12 Inhibition (%) |
| --- | --- | --- | --- | --- | --- | --- | --- |
| **1** | 70.1 | **81** | 84.8 | **161** | 64.8 | **241** | 99.4 |
| **2** | 59.6 | **82** | 102 | **162** | 53.8 | **242** | 110.9 |
| **3** | 68.8 | **83** | 99.1 | **163** | 65.7 | **243** | 102.7 |
| **4** | 77.2 | **84** | 91.3 | **164** | 78.2 | **244** | 93.4 |
| **5** | 71 | **85** | 104.2 | **165** | 99.9 | **245** | 103.9 |
| **6** | 71.4 | **86** | 86.5 | **166** | 86.8 | **246** | 98.3 |
| **7** | 69.8 | **87** | 93.7 | **167** | 72.2 | **247** | 92.2 |
| **8** | 61.9 | **88** | 74.4 | **168** | 97.6 | **248** | 71.8 |
| **9** | 62.8 | **89** | 111.2 | **169** | 70.4 | **249** | 106.8 |
| **10** | 66.6 | **90** | 96.2 | **170** | 65.1 | **250** | 96.8 |
| **11** | 56.2 | **91** | 89.7 | **171** | 79.3 | **251** | 93.1 |
| **12** | 72.4 | **92** | 30.8 | **172** | 94.2 | **252** | 85.3 |
| **13** | 87.7 | **93** | 90.5 | **173** | 75.3 | **253** | 90.8 |
| **14** | 82.7 | **94** | 96.8 | **174** | 95.4 | **254** | 95.3 |
| **15** | 85.9 | **95** | 99.9 | **175** | 91.8 | **255** | 100.8 |
| **16** | 88 | **96** | 99.2 | **176** | 96.1 | **256** | 100.6 |
| **17** | 86.1 | **97** | 95.8 | **177** | 72.1 | **257** | 97.8 |
| **18** | 83 | **98** | 84 | **178** | 66 | **258** | 87.9 |
| **19** | 83.7 | **99** | 102 | **179** | 67.5 | **259** | 110.3 |
| **20** | 78.1 | **100** | 72.1 | **180** | 56.2 | **260** | 54.7 |
| **21** | 77.6 | **101** | 92.9 | **181** | 95.2 | **261** | 92 |
| **22** | 84 | **102** | 86.2 | **182** | 68.1 | **262** | 90.1 |
| **23** | 81.6 | **103** | 101.5 | **183** | 63.1 | **263** | 99.3 |
| **24** | 85.3 | **104** | 98.5 | **184** | 70.6 | **264** | 97.2 |
| **25** | 87.9 | **105** | 96.8 | **185** | 77.5 | **265** | 101.7 |
| **26** | 81.1 | **106** | 102 | **186** | 84.9 | **266** | 103.3 |
| **27** | 76.6 | **107** | 116.2 | **187** | 74.4 | **267** | 105 |
| **28** | 67.6 | **108** | 98.5 | **188** | 70.1 | **268** | 104.1 |
| **29** | 51.9 | **109** | 138.5 | **189** | 57.5 | **269** | 181.4 |
| **30** | 89.1 | **110** | 85 | **190** | 94.8 | **270** | 108.5 |
| **31** | 77.5 | **111** | 81.9 | **191** | 79.8 | **271** | 111.3 |
| **32** | 80.8 | **112** | 164.2 | **192** | 91 | **272** | 252.1 |
| **33** | 69.3 | **113** | 108.8 | **193** | 79.5 | **273** | 83.2 |
| **34** | 43.7 | **114** | 99.8 | **194** | 82.5 | **274** | 107.3 |
| **35** | 82.7 | **115** | 84.5 | **195** | 95.3 | **275** | 81.1 |
| **36** | 77.3 | **116** | 82 | **196** | 84.1 | **276** | 74.2 |
| **37** | 83.6 | **117** | 103.9 | **197** | 88.5 | **277** | 91.5 |
| **38** | 76.6 | **118** | 159 | **198** | 76.1 | **278** | 82.4 |
| **39** | 104.2 | **119** | 125.2 | **199** | 99.4 | **279** | 76.3 |
| **40** | 70.8 | **120** | 115 | **200** | 67.1 | **280** | 134.5 |
| **41** | 66 | **121** | 94.6 | **201** | 95.3 | **281** | 100.2 |
| **42** | 80.9 | **122** | 94.8 | **202** | 73.5 | **282** | 102.1 |
| **43** | 30.2 | **123** | 89.2 | **203** | 83.1 | **283** | 97.7 |
| **44** | 78.2 | **124** | 85 | **204** | 65.5 | **284** | 91.8 |
| **45** | 80.6 | **125** | 96.7 | **205** | 75.5 | **285** | 97 |
| **46** | 83.1 | **126** | 91.2 | **206** | 76.6 | **286** | 93.5 |
| **47** | 70.7 | **127** | 95.6 | **207** | 64.8 | **287** | 103 |
| **48** | 107.3 | **128** | 69.8 | **208** | 113.1 | **288** | 74.6 |
| **49** | 84.4 | **129** | 95 | **209** | 88.5 | **289** | 100.9 |
| **50** | 76.8 | **130** | 35.7 | **210** | 66.1 | **290** | 88.1 |
| **51** | 31.6 | **131** | 91 | **211** | 91.1 | **291** | 65 |
| **52** | 63.9 | **132** | 91 | **212** | 51.8 | **292** | 89.2 |
| **53** | 70 | **133** | 90.9 | **213** | 60.4 | **293** | 93.5 |
| **54** | 82.2 | **134** | 83.6 | **214** | 68.9 | **294** | 85.2 |
| **55** | 81.4 | **135** | 90.6 | **215** | 68 | **295** | 92.4 |
| **56** | 83.1 | **136** | 84.7 | **216** | 72.4 | **296** | 87 |
| **57** | 75.6 | **137** | 79.2 | **217** | 90.5 | **297** | 81.5 |
| **58** | 75.6 | **138** | 82.4 | **218** | 66.4 | **298** | 86.9 |
| **59** | 57.7 | **139** | 82.3 | **219** | 80.4 | **299** | 88.2 |
| **60** | 74.6 | **140** | 81.3 | **220** | 69.8 | **300** | 84 |
| **61** | 97.2 | **141** | 78.6 | **221** | 98.5 | **301** | 83.5 |
| **62** | 106 | **142** | 82.1 | **222** | 100.9 | **302** | 83.2 |
| **63** | 91.3 | **143** | 80.3 | **223** | 100.6 | **303** | 82.3 |
| **64** | 92.2 | **144** | 79.1 | **224** | 96.4 | **304** | 84.1 |
| **65** | 92.4 | **145** | 100.7 | **225** | 102.7 | **305** | 91.6 |
| **66** | 119.2 | **146** | 96.5 | **226** | 97.9 | **306** | 88.8 |
| **67** | 96.7 | **147** | 120 | **227** | 79.2 | **307** | 118.1 |
| **68** | 90.8 | **148** | 169.7 | **228** | 85.5 | **308** | 147.5 |
| **69** | 104.1 | **149** | 89.8 | **229** | 110 | **309** | 87.8 |
| **70** | 75.1 | **150** | 198.3 | **230** | 75.1 | **310** | 141.3 |
| **71** | 97 | **151** | 92 | **231** | 93.7 | **311** | 100 |
| **72** | 84.3 | **152** | 88.5 | **232** | 92.1 | **312** | 104.2 |
| **73** | 95.8 | **153** | 73.4 | **233** | 99.3 | **313** | 122 |
| **74** | 106.1 | **154** | 33 | **234** | 121.8 | **314** | 171.8 |
| **75** | 105.7 | **155** | 81.9 | **235** | 117.3 | **315** | 91.8 |
| **76** | 98.4 | **156** | 87.8 | **236** | 102.9 | **316** | 118.1 |
| **77** | 97.8 | **157** | 94.6 | **237** | 100.4 | **317** | 167.5 |
| **78** | 98.4 | **158** | 94.9 | **238** | 110.2 | **318** | 87.8 |
| **79** | 98.1 | **159** | 81.6 | **239** | 104.3 | **319** | 92.8 |
| **80** | 89.2 | **160** | 76.5 | **240** | 94.7 | **320** | 83.3 |

**Supplementary Table 4.** Chemical structures of commercially available natural organic compounds **1**-**72**.

| **Entry** | **Structure** | **Entry** | **Structure** |
| --- | --- | --- | --- |
| **1**  Cyclocytidine HCl | 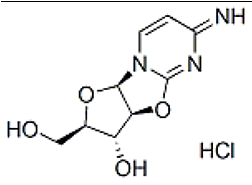 | **37**  Hesperetin | 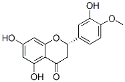 |
| **2**  (-)-Epigallocatechin Gallate | 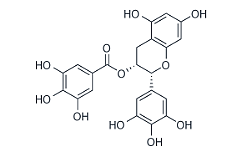 | **38**  Hesperidin | 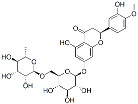 |
| **3**  D-Usnic Acid | 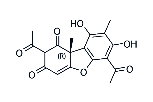 | **39**  Honokiol | 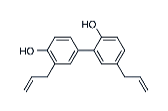 |
| **4**  3-Indolebutyric acid | 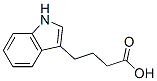 | **40**  Hyodeoxycholic | 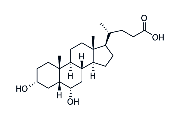 |
| **5**  4-Methylumbelliferone | 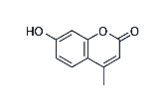 | **41**  Icariin | 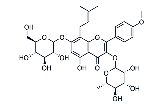 |
| **6**  Silymarin | 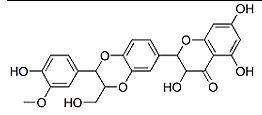 | **42**  Ndole-3-carbinol | 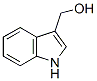 |
| **7**  Amygdalin | 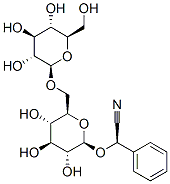 | **43**  Kaempferol | 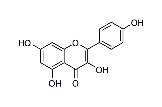 |
| **8**  Andrographolide | 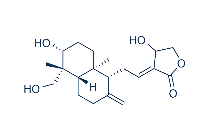 | **44**  Kinetin | 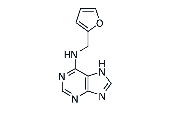 |
| **9**  Apigenin | 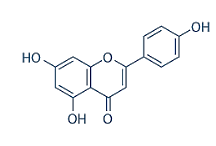 | **45**  L-(+)-Rhamnose Monohydrate | 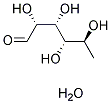 |
| **10**  Arbutin | 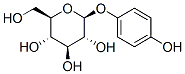 | **46**  Limonin | 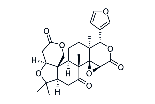 |
| **11**  Artesunate | 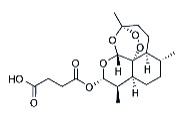 | **47**  Terminalia chebula | 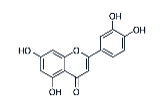 |
| **12**  Asiatic acid | 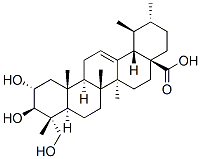 | **48**  Magnolol | 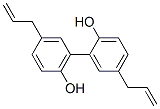 |
| **13**  Azomycin | 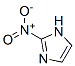 | **49**  (+)-Matrine | 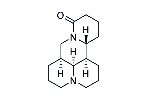 |
| **14**  Baicalein | 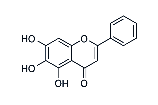 | **50**  Methyl-Hesperidin | 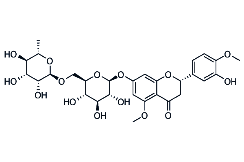 |
| **15**  Baicalin | 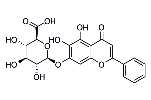 | **51**  Morin hydrate | 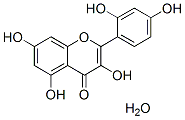 |
| **16**  Bergenin | 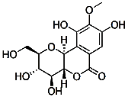 | **52**  Myricetin | 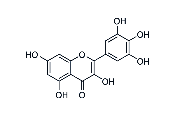 |
| **17**  Berberine chloride | 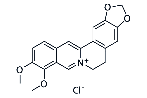 | **53**  Myricitrin | 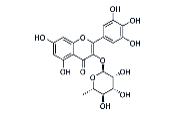 |
| **18**  Bilobalide | 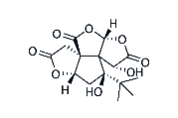 | **54**  Nalidixic acid | 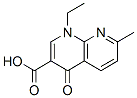 |
| **19**  Caffeic Acid | 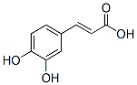 | **55**  Naringin | 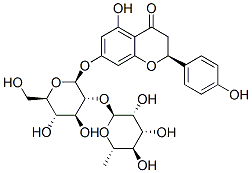 |
| **20**  Chlorogenic acid | 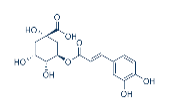 | **56**  Neohesperidin dihydrochalcone | 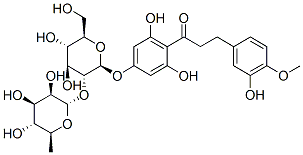 |
| **21**  Cinchonidine | 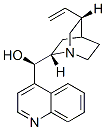 | **57**  Neohesperidin | 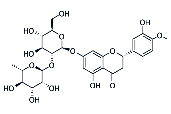 |
| **22**  Chrysin | 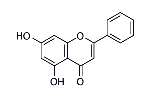 | **58**  Nobiletin | 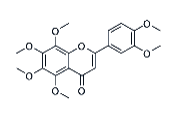 |
| **23**  Cryptotanshinone | 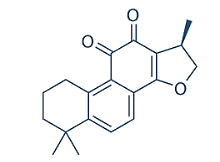 | **59**  Oleanolic Acid | 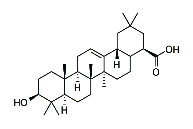 |
| **24**  Cyclosporine A | 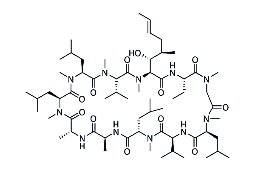 | **60**  Oridonin | 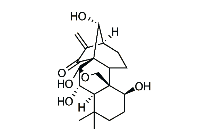 |
| **25**  Cytisine | 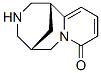 | **61**  Orotic acid | 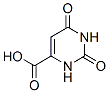 |
| **26**  Dihydroartemisinin | 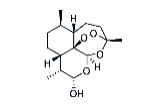 | **62**  Osthole | 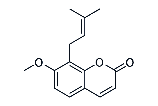 |
| **27**  DL-Carnitine HCl | 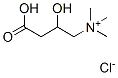 | **63**  Oxymatrine | 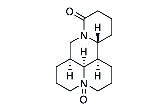 |
| **28**  Emodin | 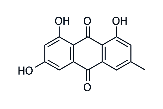 | **64**  Paeonol | 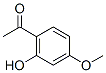 |
| **29**  Fisetin | 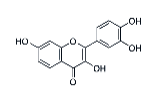 | **65**  (-)-Parthenolide | 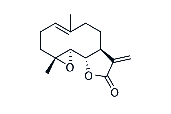 |
| **30**  Enoxolone | 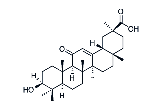 | **66**  Dihydronaringenin | 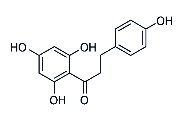 |
| **31**  Formononetin | 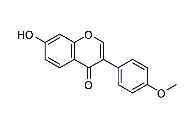 | **67**  Phlorizin | 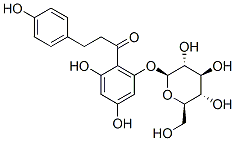 |
| **32**  Ferulic Acid | 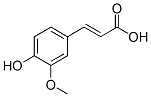 | **68**  Piperine | 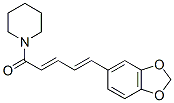 |
| **33**  Glycyrrhizin | 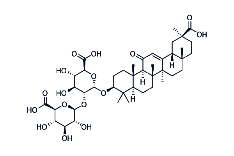 | **69**  Puerarin | 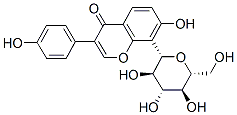 |
| **34**  Gossypol Acetate | 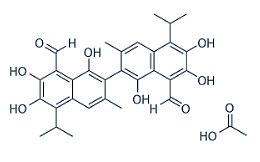 | **70**  Quercetin | 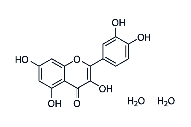 |
| 35  Gramine | 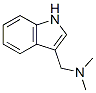 | **71**  Rutaecarpine | 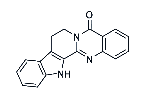 |
| **36**  Gynostemma Extract | 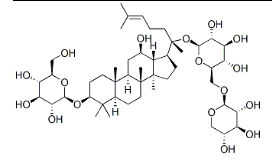 | **72**  Rutin | 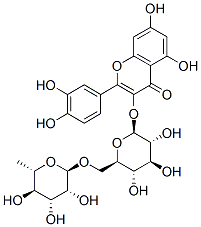 |
